# A bivalent remipede toxin promotes calcium release via ryanodine receptor activation

**DOI:** 10.1038/s41467-023-36579-w

**Published:** 2023-02-23

**Authors:** Michael J. Maxwell, Chris Thekkedam, Cedric Lamboley, Yanni K.-Y. Chin, Theo Crawford, Jennifer J. Smith, Junyu Liu, Xinying Jia, Irina Vetter, Derek R. Laver, Bradley S. Launikonis, Angela Dulhunty, Eivind A. B. Undheim, Mehdi Mobli

**Affiliations:** 1 Centre for Advanced Imaging, Australian Institute for Bioengineering and Nanotechnology, The University of Queensland, St. Lucia, QLD 4072 Australia; 2 Eccles Institute of Neuroscience, John Curtin School of Medical Research, Australian National University, Canberra, ACT 2601 Australia; 3 School of Biomedical Sciences, The University of Queensland, St. Lucia, QLD 4072 Australia; 4 Institute for Molecular Bioscience, The University of Queensland, St. Lucia, QLD 4072 Australia; 5 School of Pharmacy, The University of Queensland, Woolloongabba, Australia; 6 School of Biomedical Sciences and Pharmacy, University of Newcastle, Newcastle, NSW 2308 Australia; 7 Centre for Ecological and Evolutionary Synthesis, Department of Biosciences, University of Oslo, 0316 Oslo, Norway; 8 Present Address: Developmental and Regenerative Biology Division, Victor Chang Cardiac Research Institute, Darlinghurst, NSW 2010 Australia

**Keywords:** Peptides, Solution-state NMR, Molecular evolution, Ion transport, Ion channels

## Abstract

Multivalent ligands of ion channels have proven to be both very rare and highly valuable in yielding unique insights into channel structure and pharmacology. Here, we describe a bivalent peptide from the venom of *Xibalbanus tulumensis*, a troglobitic arthropod from the enigmatic class Remipedia, that causes persistent calcium release by activation of ion channels involved in muscle contraction. The high-resolution solution structure of φ-Xibalbin3-Xt3a reveals a tandem repeat arrangement of inhibitor-cysteine knot (ICK) domains previously only found in spider venoms. The individual repeats of Xt3a share sequence similarity with a family of scorpion toxins that target ryanodine receptors (RyR). Single-channel electrophysiology and quantification of released Ca^2+^ stores within skinned muscle fibers confirm Xt3a as a bivalent RyR modulator. Our results reveal convergent evolution of RyR targeting toxins in remipede and scorpion venoms, while the tandem-ICK repeat architecture is an evolutionary innovation that is convergent with toxins from spider venoms.

## Abstract (web summary)

Insect toxins with tandem repeats of neurotoxin domains have been found with enhanced receptor avidity. Here, the authors describe a bivalent toxin from remipede venom that targets ryanodine receptors, a rare target for animal venoms.

## Introduction

Natural products are an important source of bioactive molecules, used widely in the development of therapeutics and as selective ligands in studies of physiology^1^. Among natural products, venom-derived peptide toxins have emerged as important ion channel ligands due to their high potency and selectivity^2^. Ion channels are the fastest cellular signaling elements, and it is therefore not surprising that many venomous animals have convergently evolved to target these proteins to elicit a rapid physiological response^2,3^.

Venom peptides that target ion channels typically fold into highly ordered tertiary structures — often referred to as domains — that are stabilized through the formation of multiple intra-molecular disulfide bonds^4^. The stabilizing disulfide bonds enable hypervariability of non-cysteine residues which diversify and refine toxin function, resulting in toxin-receptor interactions with exceptionally high specificity and potency^5^. Indeed, through evolutionary processes, activity-based structural refinement has led to multiple independent lineages of toxins that target the same receptors, resulting in structural convergence of these peptides^6^. Exploring the interactions of these relatively large (peptidic) ligands with their receptors has revealed them to stabilize specific receptor states, thereby improving our understanding of ion channel function and in some cases providing leads for developments of new therapeutics and agrochemicals^7–9^. One such domain that is commonly found in animal venoms is the inhibitor cystine knot (ICK), which consists of an anti-parallel β-sheet stabilized by cysteines in a pseudo-knot arrangement that makes it both stable and highly tolerant to inter-cysteine sequence variability^5,10^.

There is emerging evidence that some venomous organisms produce toxins with multiple repeating peptide domains that have a multivalent mode-of-action^11^. Several two-domain ‘tandem repeat toxins’ have been described that contain two ICK domains, all from the venoms of (distantly related) spiders^12–14^. While the two “double-knot” toxins that have been studied in detail target different receptors—one is a capsaicin receptor (TRPV1) agonist while the other is an acid sensing ion channel (ASIC1a) inhibitor^12,13^ — they both display remarkable receptor avidity driven by a bivalent receptor binding mode^15^. The evolution of tandem repeats of peptide domains adds an intriguing new dimension to venom evolution, where the emerging quaternary structure can further modulate the potency and specificity of existing toxin domains. It also raises the question as to whether such multidomain toxins are evolutionary rarities, or whether animal venoms are indeed potentially rich sources of this emerging class of bivalent and pharmacologically interesting peptides.

Here, we present the characterization of a double-knot toxin from the venom of a remipede, *Xibalbanus tulumensis*, and show that it targets ryanodine receptors (RyRs). Remipedes are a recently discovered class of crustaceans^16^, and while proteomic studies have revealed their venoms to contain high toxin diversity, little is known of the evolution or pharmacology of these toxins^17^. We provide structural and functional characterization of φ-Xibalbin3-Xt3a (henceforth Xt3a), showing that it assumes a well-structured tandem domain arrangement — including an unusual, disordered N-terminal tail — and that it displays remarkable potency and avidity towards RyRs. Our results provide insight into remipede venom pharmacology and provide intriguing examples of convergent evolutionary innovation of molecular structure and function across multiple venomous lineages.

## Results

### Xt3a identified as a putative tandem repeat Ryanodine receptor modulator

**Fig. 1:**
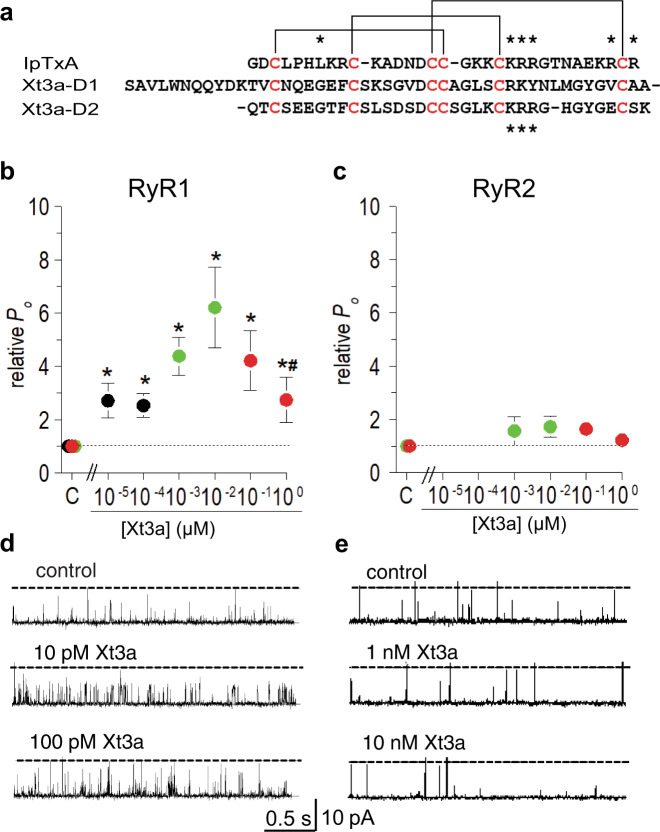
Xt3a activates ryanodine receptors (RyRs) in a similar way to other toxins in the calcin family. **a** Multiple sequence alignment of the individual Xt3a domains with Imperatoxin-A (IpTxA). The disulfide framework of IpTxA is shown above the alignment. Highlighted in red are the cysteine residues, the asterisks above IpTxA indicates residues critical for RyR activity, and the asterisks below Xt3a-D2 indicate shared critical residues. **b**, **c** The relative open probability (relative *P*_*O*_) of RyR1 and RyR2 channels as indicated respectively, with varying [Xt3a]. Data are colour coded to indicate data obtained at 10 pM then 100 pM (black: RyR1, *n* = 7, *P* = 2.03E-02 then *P* = 3.12E-03), 1 nM then 10 nM (green: RyR1 *n* = 7, *P* = 3.75E-04 then *P* = 3.76E-03;RyR2, *n* = 6, *P* = 3.80E-01 then *P* = 6.82E-02) or 100 nM then 1 µM (red: RyR1 *n* = 10, *P* = 1.00E-02 then *P* = 2.69E-02; RyR2 *n* = 7, *P* = 1.01E-01 then *P* = 1.51E-01). Asterisks indicate values that are significantly different from control. The hash symbol indicates a significant difference between average relative *P*_*O*_ between 1 μM and 10 nM (*n* = 10 and *n* = 8, *P* = 4.03E-02). The symbols show mean ± SEM and n refers to the number of observations included in the mean, with observations at +40 mV and −40 mV included for each channel, except where data from a channel at one potential was excluded (see “Methods”). Significance was determined using a two sided Students *t*-test. **d**, **e** Show 3 s from: a RyR1 channel before and after exposure to 10 pM then 100 pM Xt3a, a RyR2 channel before and after exposure to 1 nM then 10 nM Xt3a.

The remarkable pharmacology of tandem-repeat toxins led us to devise a data-mining approach to identify uncharacterised bivalent toxins. First, we extracted all secreted protein sequences from UniProtKB; a database of protein sequence information^18^. Next a Secreted Cysteine-rich REpeat Protein (SCREP) processing algorithm (SPA) was employed to identify the existence of repeating cysteine-rich domains, by segmenting individual sequences and performing an iterative BLAST function^19^, thereby locating homologous intra-sequence regions^11^. The resulting output contains a large amount of putative SCREPs (121,856) sequences with a high degree of structural and taxonomic diversity^11,20^. We then used an annotated dataset of venom peptides from UniProt known as Tox-Prot^21,22^, to further identify putative bioactive SCREPs based on their sequence identity to known toxins, resulting in 10,176 putative ion channel-targeting SCREPs showing similarity to 494 different single domain toxins. Among these we identified three double-ICK SCREPs (Xt1a-3a), which were originally identified by analysis of protein sequences translated from venom gland transcriptomics of the remipede *Xibalbanus tulumensis*^17^. Xt3a, an 82-residue peptide, was selected due to the high level of sequence similarity it shares with imperatoxin-A (IpTxA), a member of the calcin family of scorpion derived ICK toxins. The calcins are known to modulate activity of the intracellularly located ryanodine receptors (RyR), which are responsible for Ca^2+^ release within both skeletal and cardiac muscle^23,24^, as well as in many neuronal cells^25^. The C-terminal domain of Xt3a (Xt3a-D2) contains 3 of 6 analogous residues previously identified as critical for the activity of IpTxA (Fig. 1a), an important feature shared across all calcins^26,27^.

### Xt3a is a potent and selective RyR modulator

Xt3a was produced recombinantly as an MBP fusion protein in *E. coli,* using a periplasmic expression method^28^, and after cleavage from the fusion tag, HPLC purification revealed a dominant, oxidatively folded, toxin isoform. The purified peptide was used in single channel electrophysiology recordings where RyR1 or RyR2 channels were inserted into a lipid bilayer as described previously^29^. Average channel open probabilities (*P*_*O*_) of RyR1 (relative to baseline levels prior to the addition of toxin) demonstrate significant changes in all Xt3a concentrations tested over the range 10 pM to 1 µM (Fig. 1 and Supplementary Figs. 1–4). High affinity activation of RyR1 at Xt3a concentrations up to 10 nM led to an increase in full conductance channel openings (Fig. 1). At higher Xt3a concentrations (0.1–1 µM), we observed a decline in *P*_*O*_ by ~50% compared with 10 nM Xt3a and a reduction in full conductance openings (Supplementary Figs. 1–2), indicating a secondary inhibitory effect. This secondary effect of reducing channel openings from full to half conductance is reminiscent of the IpTxA induced RyR1 substate^29^. In contrast to the significant effects of Xt3a towards RyR1, RyR2 channels did not show any consistent changes in openings to the maximum conductance at Xt3a concentrations ranging from 1 nM to 1 μM (Fig. 1). RyR2 channels exhibited both low and high base level activity in the presence of 1 μM Ca^2+^ concentrations (Supplementary Figs. 1–2). However, neither the low or high activity channels (Supplementary Fig. 1c and d, respectively) responded in any significant manner to exposure to Xt3a, with the exception of a weak trend towards increased activity with 1 nM and 10 nM concentrations (Fig. 1c). These data indicate presence of low affinity inhibition that partially masks the high affinity activation, suggesting two separate binding sites. An additional effect observed was a voltage-dependent promotion of channel substate activity by Xt3a – being greater at −40 mV (Supplementary Fig. 3) than at +40 mV (see Fig. 1 and Supplementary Figs. 1–3) and greater in the presence of nanomolar concentrations of Xt3a in RyR1 than in RyR2; a characteristic effect previously observed with both IpTxA and ryanodine^29,30^.

The specificity of Xt3a was assessed by performing assays against common toxin targets. Addition of Xt3a (3 µM) did not elicit an increase in intracellular Ca^2+^ in SH-SY5Y neuroblastoma cells expressing a range of ion channels and G protein-coupled receptors^31–34^ (Supplementary Fig. 5). Moreover, Xt3a (3 µM) did not inhibit Ca^2+^ responses elicited by activation of endogenously expressed α7 nAChR, α3-containing nAChR, Ca_V_1.3, Ca_V_2.2, Na_V_1.2 and Na_V_1.7 channels (Supplementary Fig. 5b).

### Xt3a is a double-ICK peptide with a semi-rigid domain linker

**Fig. 2:**
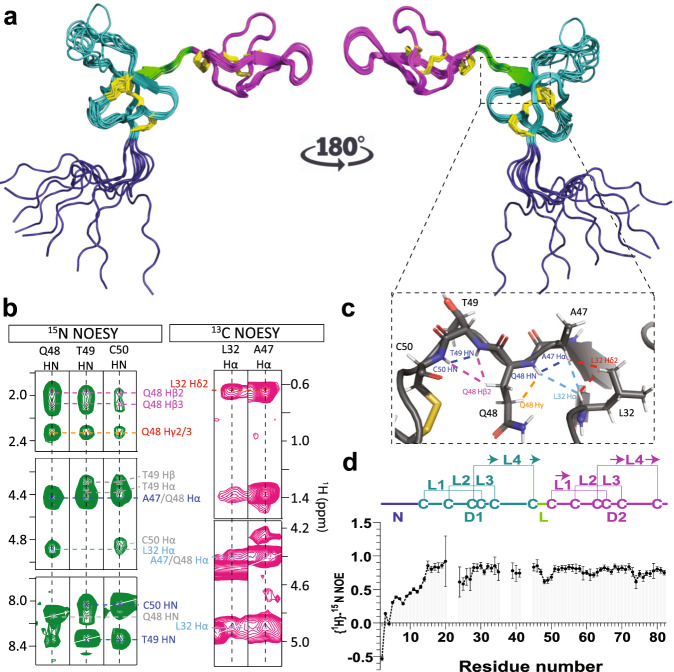
Solution structure of the double-knot remipede toxin Xt3a. **a** NMR derived Xt3a structural ensemble, front and back (PDB ID code 7RZ3). The N-terminal domain (D1) is shown in cyan, C-terminal domain (D2) in magenta, the connecting linker region in green, the extended N-terminal region in blue, and the disulfide bond connectivity in yellow. **b** ^15^N and ^13^C NOESY cross-peaks corresponding to sidechain distance restraints that stabilize the inter-domain linker “m”-turn. **c** Atomic structure of the linker region. Dashed lines indicate the distance restraints derived from the NOESY cross-peaks shown in (**b**). **d** ^1^H-^15^N heteronuclear NOE ratios indicating a disordered N-terminal tail and a less dynamic second domain (errors based on spectral noise). No amide resonances were observed between residue positions 21-23, 36-38, and 42-44. A schematic of Xt3a architecture with domains colored according to (**a**) is shown above the graph, highlighting the N-terminal region (N), each disulfide rich domain (D1, D2), and the connecting linker region L. The cysteine connectivity is shown with horizontal brackets, and arrows indicating the anti-parallel β-sheet secondary structure.

The double-ICK architecture of Xt3a was confirmed by producing a uniformly ^13^C and ^15^N isotope enriched Xt3a sample, which was used to generate an exhaustive set of heteronuclear NMR experiments (see Materials and Methods). The high-resolution structure, solved using ~1500 experimental constraints (Supplementary Table 1), reveals two independently folded domains separated in space by a peptide linker. The two domains adopt a back-to-back orientation (Fig. 2a) – where the two ICKs are translated 180° about two orthogonal axes. This quaternary domain configuration does not result in clear inter-domain dipolar couplings (NOEs); however, the fold was confirmed by a network of clear dipolar couplings along the linker, further stabilized by a covalent bond formed between a cysteine flanking the linker region and a cysteine in the second domain, forming an “m” shaped turn along the backbone (Fig. 2b and c).

The structure calculations also revealed that the N-terminal region is disordered (residues 1–12) (Fig. 2d). This region contains several hydrophobic residues (A2-V3-L4-W5) perhaps indicating a potential role for membrane binding. We therefore performed NMR titration experiments to assess the binding of ^15^N Xt3a against cyclized nanodiscs containing zwitterioninc lipids. We found no evidence of specific binding to the bilayer, while several regions appear to weakly and non-specifically bind to the nanodiscs (Supplementary Fig. 6). These regions may point to potential hydrophobic binding interfaces involved in protein-protein interactions, most notably involving V26. This is contrary to outcome of similar experiments using known membrane active peptides^35,36^.

The indication of a dynamic N-terminal region and the presence of a rigid and short inter-ICK linker prompted us to further investigate the dynamics of the peptide using ^15^N spin relaxation studies (Fig. 2d and Supplementary Fig. S7). The N-terminal region shows clear disorder as indicated by low or negative heteronuclear NOE ratios. The linker region shows NOE values closer to but lower than those of the structured regions of the peptide, indicating that the linker is not completely rigid. This observation makes the Xt3a linker more rigid than that of DkTx but less so than the linker of Hi1a. These results indicate that the Xt3a linker more closely resembles the linker of Hi1a than DkTx^12,13^. Furthermore, we find that while the N-terminal ICK-domain appears to be overall less ordered (on a fast time-scale) than the C-terminal ICK domain, the R1 and R2 rates are in a similar range for the two domains, indicating that these share a common correlation time – consistent with the short and semi-rigid linker. The N-terminal tail, on the other hand, appears to reorient in solution largely independently of the other two domains, consistent with the observed disorder.

### Xt3a induces a persistent RyR response

**Fig. 3:**
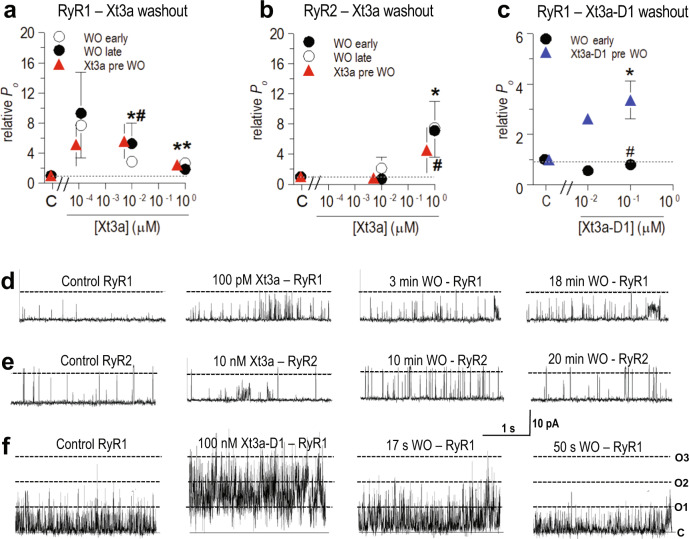
Electrophysiological response after removal of Xt3a and Xt3a-D1. **a**, **b** Average relative *P*_*O*_ during exposure of RyR1 and RyR2 channels to Xt3a (red triangles) and after removal of Xt3a (washout, WO) by perfusion with solution lacking Xt3a (after 3–10 min, ‘early’, filled circles and after 15–30 min, ‘late’, open circles). **c** Average relative *P*_*O*_ of RyR1 channels exposed to Xt3a-D1 (blue trianlges) and after washout (filled circles, 30–60 s after Xt3a-D1 WO). The data at 10 nM shows the relative *P*_*O*_ for an individual channel and the data at 100 nM show average relative *P*_*O*_ of 4 observations from 3 channels exposed to Xt3a-D1. * indicates significant difference from control according to the *t*-test. # indicates a significant difference according to the sign test in (**a**) and (**b**) or a significant decline after washout in (**c**). **a** RyR1-Xt3a. For 10^−4^ µM, *n* = 4: pre, *P* = 1.01E-01; early, *P* = 1.68E-01; late, *P* = 1.71E-01. For 10^−2 ^µM Xt3a, *n* = 4: pre, *P* = 4.67E-02; early, *P* = 3.24E-02; late, *P* = 4.68E-03. For 1 µM, *n* = 4; pre, *P* = 4.67E-02; early *P* = 3.77E-03; late *P* = 1.17E-01. **b** RyR2-Xt3a. For 10^−2 ^µM, *n* = 4: pre, *P* = 7.57E-01; early, *P* = 4.32E-01; late, *P* = 4.17E-01. For 1 µM, *n* = 5: pre, *P* = 1.35E-01; early, *P* = 4.68E-03; late, 4.08E-02. **c** RyR1-Xt3a-D1. For 10^−1 ^µM, *n* = 4, *P* = 5.66E-03 compared to control, or *P* = 2.16E-02 for WO compared to pre. The symbols show mean ± SEM and *n* refers to the number of observations included in the mean. Significance was determined using a two sided Students *t*-test for the subset of RyR2 channels maintained through Xt3a washout. A 1-tailed paired *t*-test was used with the subset of RyR1 maintained through Xt3a washout shown in Fig. 3A (*n* = 4). **d**, **e**, **f** 2 single low activity channel recordings in (**d**) and (**e**) and one recording of multiple high activity channels in (**f**). Records from each channel were obtained under control conditions (column 1) and after exposure to the peptides as indicated. The parallel lines (labelled C, O1, O2, O3 and O4 in (**f**) column 4) indicate the closed level and open levels when 1, 2 or 3 channels respectively open simultaneously.

The two previously reported venom peptides with a tandem repeat ICK structure — the spider toxins DkTx and Hi1a — both display high avidity, evidenced by persistent activity during washout from their respective ion channel targets, TRPV1 and ASIC1a respectively. To examine if Xt3a displays a similar high avidity towards RyR channels, a series of electrophysiology washout experiments were performed to determine the reversibility of Xt3a effects. RyR1 channel recordings were taken before and during exposure to 100 pM, 10 nM and 1 μM Xt3a, then “early” (2 to 3 min) and “late” (15 to 23 min) after washout (Fig. 3a). Notably, no significant washout of Xt3a effect was observed, with activity remaining much higher than the control throughout the washout period. It is also notable that sub-state activity increased during the washout period and was maintained for as long as the bilayer lasted.

With RyR2 there was no significant change in activity during exposure to the toxin as discussed above. Surprisingly, activity did appear to increase somewhat during washout (Fig. 3). Activity in some RyR2 channels increased shortly after washout and then declined at longer times as seen in the first RyR2 channel (Supplementary Fig. 8) after washout of Xt3a at 10 nM. In other RyR2 channels, e.g., the second RyR2 channel recordings (Supplementary Fig. 8), activity did not change significantly at short times but then increased after longer washout periods following exposure to Xt3a at 1 μM. The increased activity during washout could be attributed to the unmasking of RyR2 activation by removal of a weak inhibitory effect of Xt3a binding to a low affinity site.

### Xt3a is a bivalent toxin that enhances Ca^2+^ leak from the sarcoplasmic reticulum

The persistent Xt3a response (high avidity) suggests a bivalent mode of action. We, therefore, produced each domain of Xt3a (sequences as shown in Fig. 1a); Xt3a-D1 and Xt3a-D2, as well as IpTxA recombinantly as SUMO fusions, in the above-described expression system and purified by rpHPLC (Supplementary Figs. 9, 10). Electrophysiology experiments were carried out to determine the potency of each peptide. While both IpTxA and Xt3a-D1 showed potent (pM to nM) activation of RyR1 channels, Xt3a-D2 was inactive at all concentrations tested (Supplementary Fig. 11). It is known that the activity of IpTxA is reversible^29^, so we next performed washout experiments to determine if the activity of Xt3a-D1 was also reversible. The results show a rapid return to control conditions after less than 1 minute of washout. Taken together these results indicate that the activity of Xt3a is largely due to the first domain (Xt3a-D1) while the presence of the second domain (Xt3a-D2) makes this activity persistent.

**Fig. 4:**
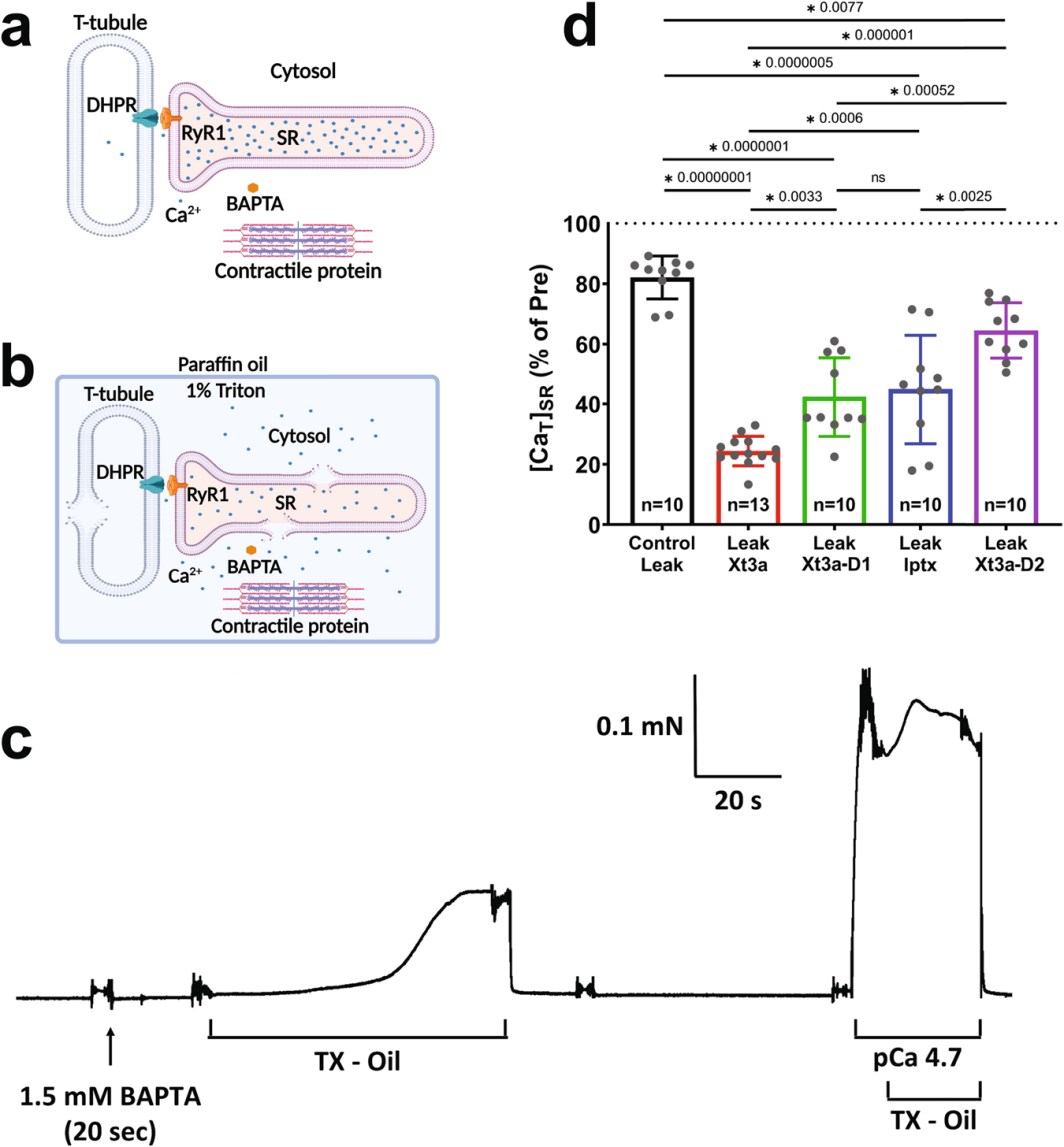
Xt3a potently and bivalently activates ryanodine receptors. **a** Schematic diagram of a mechanically-skinned muscle fiber. The sarcolemma is removed, resulting in the t-system sealing off. This preparation allows the manipulation of the intracellular conditions of the fiber in a very rapid and controlled way. **b** Following the pre-equilibration of the myoplasm with the high-affinity Ca^2+^ buffer BAPTA, the muscle fiber is plunged in an emulsion of paraffin oil and Triton-X100 (TX-Oil) which lyse the SR and other internal membranes, releasing the stored Ca^2+^ into the myoplasm, where it remains trapped by the surrounding paraffin oil-Triton emulsion. Thus, the amount of Ca^2+^ that had been present in the fiber can be subsequently calculated from the level of force produced upon the lysing process and taking into account the exact [BAPTA] present within the fiber space. **c** A mechanically-skinned fiber segment from a mouse EDL with endogenous resting Ca^2+^ content was briefly equilibrated with 1.5 mM free BAPTA and when lysed in TX-Oil produced ~35% of the maximal force. **d** Relative amount of total Ca^2+^ left in the SR ([Ca_T_]_SR_) following the 1 min leak protocol with or without 10 nM of either Xt3a, Xt3a-D1, IpTxA or Xt3a-D2 present in the leak solution. Values are presented as mean ± SD, overlayed with corresponding dot plot, with *n* denoting the number of fibers examined, and are expressed relative to [Ca_T_]_SR_ before the leak (Pre). “*P < 0.01” (exact values are depicted in the figure) indicates value is significantly different than other conditions (One-way ANOVA). Only treatment with Xt3a-D1 and IptxA does not give a significant difference (*P* = 0.98).

To further test the activity in a more native environment, we employed a muscle fiber experiment^37–39^. Here, the amount of Ca^2+^ leakage out of the sarcoplasmic reticulum (SR) in mouse extensor digitorum longus (EDL) fibers is examined. To do this, we determined the total SR Ca^2+^ content ([Ca_T_]_SR_) of skinned fibers before and after a 1–min leak protocol (control leak) using a previously established membrane-lysis method, which measures the force response to Ca^2+^ liberated from lysed membrane compartments by a triton-oil emulsion (Fig. 4a, b). Experiments were also carried out with either 10 nM of Xt3a, Xt3a-D1, Xt3a-D2, or IpTxA present in the leak solution, using exactly the same procedures and solutions. It was found that [Ca_T_]_SR_ following the control leak was significantly depleted by 18 ± 7%. Importantly, we found that the presence of 10 nM of any of the four peptides in the leak solution depleted significantly more [Ca_T_]_SR_ (by 76 ± 5%, 58 ± 13%, 36 ± 9%, and 55 ± 18% for Xt3a, Xt3a-D1, Xt3a-D2, and IpTxA, respectively). These results indicate that the SR Ca^2+^ leakage in the presence of the peptides was significantly greater than the control leak alone. The efficiency of the peptides at a concentration of 10 nM to increase the SR Ca^2+^ leakage was in the following order: Xt3a > Xt3a-D1 ~ IpTxA > Xt3a-D2 (Fig. 4d). These results are consistent with the electrophysiology recordings described above.

### Evolution of a family of double calcins

To explore the evolutionary origin of Xt3a, we first performed a BLAST search against the venom gland and body transcriptomes of *X. tumulensis* using the sequences of both the full-length and single domains. These searches returned two distinct forms of putative ICK peptides: double-domain ICK peptides from the peptide family named xibalbin family 3, where each domain contained three disulfides, and single-domain putative ICK peptides from xibalbin family 1 that contain an additional disulfide that presumably stabilises loop four. Of these, only single-domain ICKs were detected in both the venom gland and body transcriptomes, indicating that the double-ICK form is exclusive to the venom. We next searched UniProtKB and the NCBI non-redundant database using BLAST. However, these BLAST searches returned no pancrustacean hits, and we therefore used jackhmmer to search all arthropod sequences contained within UniProtKB. Searching with individual ICK domains from xibalbin family 3 returned 137 pancrustacean hits, of which 53 were ICK and none were multidomain ICK. Searching with the full-length Xt3a returned only six pancrustacean hits, none of which were double-domain ICK sequences. Thus, while double-ICK peptides have previously been detected in spiders, Xt3a and the remaining members of xibalbin family 3 appear to represent a convergent class of such peptides from Pancrustacea.

**Fig. 5:**
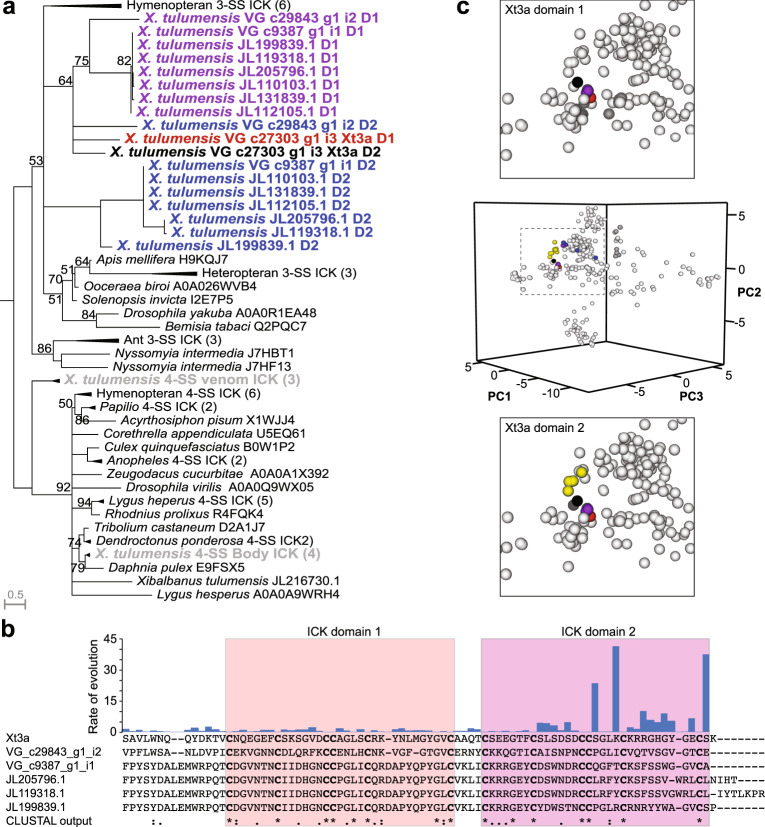
Evolution of Xt3a. **a** Maximum likelihood (ML) phylogenetic tree of pancrustacean ICK domains suggests that Xt3a most likely arose through the duplication of the ICK-encoding part of an ancestral 3-SS gene. Bootstrap support values below 95 are shown at each split and branches with less than 50 bootstrap support are collapsed into multifurcations. **b** Alignment and estimation of evolutionary rates suggests domain two has evolved faster than domain one. Site-specific substitution rates by ML are shown in the bar chart above the sequence alignment, while Clustal-format indicators of overall sequence conservation across the aligned sequences are shown below the alignment. **c** PCA analysis of the biophysical properties of each domain of Xt3a aligned with all other remipede ICK and functionally annotated ICK peptides in UniProtKB (middle). The second domains of the remipede double ICK peptides (blue) are more dispersed than the first domains (purple). While both Xt3a ICK domains are similar to the first domains from the other remipede double ICKs (purple), domain one (red; top) is similar to several voltage gated sodium channel toxins (dark grey; top), and domain two is structurally more similar to calcins (black; bottom).

Aligning the identified ICK domains and reconstructing their phylogenetic relationship by maximum likelihood confirmed that Xt3a and xibalbin family 3 did not evolve from the only single-domain ICKs detected in the transcriptomes, as evidenced by the well-supported monophyletic clades of three- and four-disulfide ICKs (Fig. 5a; Supplementary Fig. 12; Supplementary Data 1 and Supplementary Data 2). Although we were unable to completely resolve the phylogenetic relationships between the domains of xibalbin family 3, our results indicate that they probably evolved through a single domain duplication of a three-disulfide ICK precursor that has since been lost. The lack of resolution is also reflective of the variable rates of evolution between the two domains, with domain 2 containing several rapidly evolving sites (Fig. 5b and Supplementary Table 2). To further explore the domain-specific evolution of the xibalbin family 3 ICK domains, we aligned the individual domains to all functionally annotated ICK toxins in UniProtKB. We then compared the biophysical properties of each ICK domain by performing a PCA on the resulting matrix and projecting each sequence in a three-dimensional sequence space consisting of the first three principal components (Fig. 5c, middle). This approach reinforced the observed convergence between Xt3a-D2 and the calcin scorpion toxin family (Fig. 5c, and Supplementary Table 3): Calcins account for five of the ten physicochemically most similar ICK domains to Xt3a-D2, which is about as similar to the closest calcin (Imperacalcin, P59868) as it is to Xt3a-D1. In contrast, only one calcin is among the ten most similar sequences to Xt3a-D1 (10th most similar, BmCa.1, Q8I6X9). Taken together, these results suggest that xibalbin family 3, represented here by Xt3a, emerged in remipedes through a domain duplication event and then underwent functional diversification through whole-gene duplication and domain-selective evolution.

## Discussion

The structural and functional studies presented here reveal the remipede toxin, Xt3a, to be a double-knot toxin in the calcin family and a potent, specific, and avid ryanodine receptor activator. Xt3a is the fourth naturally occurring tandem repeat ICK peptide reported, and the third where the structure and function has been elucidated. While each of these three peptides target a different ion channel (DkTx with TRPV1, Hi1a with ASIC1a and Xt3a with RyR1), each display a bivalent mode-of-action, evident in a remarkable enhancement of avidity associated with their dimeric structures. In Xt3a, we observe persistent activity after washout in our single-channel recordings for as long as the bilayers lasted. In contrast, when only one of the two domains is present we see a loss of this persistent activity, supporting a multivalent binding mode. Analysis of the structure-activity data using isolated domains of Xt3a, suggests that the first domain of the peptide (Xt3a-D1) causes an increase in channel opening while the second domain (Xt3a-D2) is required to make this activity persistent, likely by binding to an adjacent receptor site. Avidity is a sought-after property of pharmaceutical drugs, leading peptide chemists to design dimeric synthetic peptides (homo or hetero-dimeric), to mimic the behavior of these natural toxins^40–42^. These investigations have had mixed results with either no gain in avidity observed or presence of avidity that cannot be linked to bivalent receptor engagement.

The source of this naturally evolved bivalency is likely a combination of individual domain sequences that target adjacent receptor sites, as well as a linker sequence to spatially orient the two domains in a defined quaternary structure. The latter may further enhance the potency of the bivalent molecule by excluding non-binding orientations or stabilizing orientations that favor bivalent binding^43,44^. Thus, the linker sequence is likely to be indirectly related to the structure of the receptor (i.e., not necessarily through direct linker–receptor interactions). Indeed, there appears to be significant variation in the linkers of the double ICK peptides studied to date, leading to distinct inter-domain orientations. In DkTx, the two domains are loosely connected via a long (12-residue) linker, and in the receptor-bound state the domains are oriented in a side-to-side orientation binding to remote but equivalent binding sites in the homo-tetrameric receptor (lateral translation along one axis to superimpose the two domains)^15^. In the case of Hi1a (6-residue linker), there is a hydrophobic pocket between the two domains where a linker phenylalanine is inserted, resulting in a back-to-side orientation (a 90° rotation about one axis followed by a 180° rotation along an orthogonal axis to superimpose the two domains)^12^. The second binding site of Hi1a on ASIC1a is not known and it is therefore unclear how this orientation leads to the observed gain in avidity. Xt3a has the shortest linker (4-residues), and folds into a back-to-back orientation (two 180° rotations about orthogonal axes for superimposition of domains). The diversity of domain orientations suggests evolution of quaternary structure in multi-domain toxins to enhance receptor avidity is an emerging and unexplored dimension of venom evolution.

The confirmation that a remipede venom contains double ICK toxins is particularly interesting given the similarity of Xt3a to the calcin scorpion toxin family. First, we observe that while the ICK-motif itself is abundant across venomous species, elaboration of this through domain duplication has only been found in spiders previously^12,13^. Second, the sequence of the C-terminal domain of Xt3a falls within the calcin family of peptides that are known to target RyRs, a family of peptides that has, to date, only included sequences from scorpions^23^. Interestingly, the second domain of Xt3a, which is the most similar to calcins, is by itself only weakly active against RyR1, perhaps because it lacks three of the six residues known to be important in the function of scorpion calcins^27^ (Supplementary Fig. 13a and b). While the striking structural convergence between Xt3a-D2 and IpTxA could indicate similar binding site, it is not the mechanism responsible for the evolution of xibalbin RyR toxins, as shown by the activity of the structurally divergent Xt3a-D1 (Supplementary Fig. 13b and c). However, given that the full length Xt3a is even more potent than IpTxA, we speculate that these convergent properties enable binding of the second ICK domain of Xt3a to a second receptor site. The modulation of RyR is thus not necessarily achieved by the overlapping structural features of Xt3a and other calcins. Instead, Xt3a achieves its full potency through an evolutionary innovation that has to date only been identified in spider venoms and subsequent convergent evolution with toxins that have to date only been identified from scorpions.

It is not surprising that there are isoform-specific differences in the effects of Xt3a on RyR1 and RyR2. There are sequence and structural differences between the proteins (Supplementary Fig. 14), and many responses can differ between the isoforms, including inactivation by high cytoplasmic Ca^2+^ ^45,46^, calmodulin^47^, and IpTxA^29,48,49^, while GSTm2 slightly increases the activity of RyR1, but strongly inhibits RyR2^50^. The difference could simply indicate that the Xt3a binding site is in the vicinity of a sequence difference that alters the structure of the binding site; or  that the sequence difference alters the intramolecular pathway between the binding site and the channel gating residues, in a manner similar to disease associated mutations in the RyR1 and RyR2^51,52^.

The data presented here clearly demonstrate that application of Xt3a leads to release of calcium stores via RyR activation, however, details of the underlying mechanism remain to be elucidated. Further biochemical, biophysical, structural and functional studies are required to determine how this is achieved. Structural details of the receptor bound form of Xt3a will be particularly valuable as it will provide a rationale for the observed domain orientation and the role of the individual domains of the peptide.

Together, our findings present a remarkable example of convergent evolution across levels of molecular structure to disrupt an excitation contraction coupling mechanism for immobilizing prey that is rarely targeted by animal venoms. However, they also point to the emergence of quaternary structure in multidomain venom peptides as a general evolutionary innovation for achieving unique pharmacological properties that enable new ecological functions. Studies of these natural products will be important in rational engineering of their synthetic counterparts as therapeutic and agrochemical leads.

## Methods

### Recombinant peptide production

Recombinant expression of Xt3a, Xt3a-D1, Xt3a-D2 and IpTxA was performed using an *E. coli* expression system. A gene encoding the peptide was subcloned into an expression vector containing a coding region with poly-histidine purification tag as well as a solubility tag (MBP for Xt3a and SUMO for Xt3a-D1, Xt3a-D2 and IpTxA) with a tobacco etch virus (TEV) protease cleavage site located between the solubility tag and target peptide. The expression vector was transformed into the genetically engineered SHuffle cells which support the correct formation of disulfide bonds^28,53^. *E. coli* SHuffle cells supplemented with ampicillin were grown at 30 °C until OD_600_ = 0.8–1.2, then induced with 0.1 mM isopropyl β-D-1-thiogalactopyranoside (IPTG) and incubated overnight at 16 °C. Purification was performed using immobilized metal affinity chromatography (IMAC) and reversed-phase HPLC standard techniques as described previously^28,36^. For IMAC purification, cells were resuspended in buffer (250 mM NaCl, 25 mM Tris, pH 7.8) and lysed using sonication, the soluble cell lysate was applied to a buffer equilibrated 5 mL Ni-NTA column (Qiagen), and the fusion proteins were eluted using 250 mM imidazole. Prior to reversed-phase HPLC, cleavage using TEV protease was performed for each fusion protein. Xt3a, Xt3a-D1 and Xt3a-D2 were cleaved in a TEV/fusion-protein ratio of 1:10 in buffer containing (150 mM NaCl, 20 mM Tris, 2.5 mM GSH, 0.25 mM GSSG, pH 7.8). IpTxA was cleaved using both SUMO and TEV protease using a SUMO/TEV/fusion-protein ratio of 1:10:300 in buffer containing (150 mM NaCl, 20 mM Tris, 5 mM GSH, 0.5 mM GSSG, 0.5 M urea, pH 7.8). The cleaved peptides were then purified using a C-18 reversed-phase HPLC column. Folding of Xt3a-D1 and Xt3a-D2 was confirmed by comparison with the 1D NMR spectra of Xt3a and IpTxA was confirmed by comparison to published NMR TOCSY spectra^27^.

### Mass spectrometry

Mass analysis of target peptides was performed using matrix-assisted laser desorption/ionization-time of flight (MALDI-TOF) mass spectrometry. The matrix was prepared using a saturated stock solution of α-Cyano-4-hydroxycinnamic acid (CHCA) in acetone, mixed with (ethanol:acetone: 0.1% trifluoroacetic acid (TFA) (6:3:1)) at a 1:9 ratio. Sample preparation was done by combining the matrix solution and the HPLC purified peptide (1:2) and adding 1.5 µL of sample to a ground steel target plate. All data was collected using linear mode on a MALDI-TOF mass spectrometer (Bruker Daltonix Autoflex Speed).

### SR isolation and single RyR channel experiments

Cardiac SR was prepared from mature female crossbred sheep hearts^46^, and skeletal SR vesicles were isolated from the back and leg muscles of mature female New Zealand white rabbits^29,54^ with approval from the ANU Animal Experiment Ethics Committee Protocol Number A2015/35. Xt3a, Xt3A-D1, Xt3A-D2, and IpTxA were tested on RyR1 and RyR2 channels inserted into lipid bilayers. Solutions for vesicle incorporation contained (mM): *cis* 230 caesium methanesulfonate (CsMS); 20 CsCl; 1 CaCl_2_ and 10 tetraethylsulfamide (TES) pH 7.4 and *trans* 30 CsMS; 20 CsCl; 1 CaCl_2_ 10 TES; pH 7.4. SR vesicles routinely incorporated with the cytoplasmic surface of the SR and RyR facing the *cis* chamber. Following incorporation, 200 mM CsMS was added to the *trans* solution and the *cis* solution exchanged with recording solution containing (mM): 230 CsMS; 20 CsCl; 0.001 CaCl_2_;10 TES and pH 7.4, with [Ca^2+^] set by a [BAPTA] determined using a Ca^2+^ electrode. The cytoplasmic and luminal solutions contained 1 μM and 1 mM Ca^2+^ respectively, with symmetrical 250/250 mM CsMS. Bilayer voltage was switched between +40 mV and −40 mV (cytoplasmic voltage relative to grounded luminal solution) every 30 s. The activity of the RyR channels used here included 1 µM Ca^2+^ in the cytoplasmic solution and 1 mM Ca^2+^ in the luminal solution^29,55^. Electrophysiology experiments were analysed using Channel 2 (P.W. Gage, M. Smith, Australian National University) and Channel 3 (N.W. Laver, University of Newcastle, Australia) software, available from the authors upon request. Statistical analyses were performed using Microsoft Excel (Office 365). The channels have a maximum current of ~10 pA and an average open probability of 0.04 ± 0.01 (RyR1) and 0.009 ± 0.003 (RyR2) before exposure to Xt3a. Following most incorporation events, a single channel only was active in the bilayer and channel open probability *(P*_*o*_*)*, mean open time *(T*_*o*_*)*, mean closed time (*T*_*c*_) and open event frequency (*F*_*o*_) were measured. When 2–4 channels were active in a bilayer, *P*_*O*_ was approximated from the fractional mean current (*I’F*), i.e., mean current divided by maximum current. *I’F* is equal to *P*_*O*_ in a single channel and indicates the average *P*_*O*_ of multiple channels in a bilayer^56^. The experiments exploring the detailed actions of Xt3a on RyR1 and RyR2 were collected and analysed at +40 mV and at −40 mV (at the Australian National University – ANU). Experiments exploring the actions of Xt3a-D1, Xt3a-D2 and IpTxA on RyR1 were collected only at +40 mV (at the University of Newcastle – UN). Experiments at UN also included a minority of multi-channel experiments. The bilayer rigs, solution composition, source of channels, data acquisition and analyses methods were all identical. Parameters measured at +40 mV and at −40 mV showed similar effects of Xt3a, therefore data from both potentials were combined in the average parameter values reported under each condition. In all experiments control channel activity was recorded for 3 to 5 min before exposure to the test compound. To fully explore the actions of Xt3a, each channel was exposed to only two concentrations of Xt3a before washout in order to detect the full effect of each concentration over time periods of 5 to 10 min. Therefore, separate data sets for RyR1 were obtained for: 10 and 100 pM; 1 and 10 nM; and 0.1 and 1 µM Xt3a. Separate data sets for RyR2 were obtained for: 1 and 10 nM; and 0.1 and 1 µM Xt3a. Detailed Xt3a washout experiments were performed by perfusion of the bilayer bath with a recording solution lacking Xt3a after which RyR activity was recorded for 15 to 30 min. In experiments to assess relative activity and avidity of Xt3a-D1, Xt3a-D2, IpTxA and Xt3a (at UN), channels were exposed to each peptide for 1 to 5 min (5 min for Xt3a) followed by washout for 3–25 min (3 min for Xt3a-D1, 25 min for Xt3a) before repeating by adding the same or a different compound.

### Cell based fluorescence imaging plate reader (FLIPR) Ca^2+^ assays

SH-SY5Y human neuroblastoma cells (European Collection of Authenticated Cell Culture, Sigma Aldrich, 94030304-1VL) were cultured in RPMI media (ThermoFisher Scientific, Scoresby, Australia) supplemented with 15% foetal bovine serum and L-glutamine (1 mM) and passaged at approximately 80% confluency with TrypLE Express (Thermo Fisher, Australia). For fluorescence imaging, cells were plated on CellBIND (Corning) 384-well black-walled imaging plates at a density of 20,000–30,000 cells/well in complete media and cultured for 48 h at 37 °C/5% CO_2_.

The effects of Xt3a (3 µM) on endogenously expressed α7 nAChR, α3β2/α3β4 nAChR (referred to as α3-containing; α3 nAChR), Ca_V_1.3, Ca_V_2.2, Na_V_1.2 and Na_V_1.7 channels were assessed by fluorescence Ca^2+^ imaging in SH-SY5Y cells as previously described^33,34^. Briefly, SH-SY5Y cells were loaded with Calcium 4 no-wash dye (Molecular Devices) diluted in physiological salt solution (composition in mM: NaCl 140, D-glucose 11.5, KCl 5.9, MgCl_2_ 1.4, NaH_2_PO_4_ 1.2, NaHCO_3_ 5, CaCl_2_ 1.8, HEPES 10) for 30 min at 37 °C. A two-addition FLIPR^Tetra^ (Molecular Devices) protocol using the following settings was used to measure fluorescence responses: excitation 470–495 nm; emission 515–575 nm; excitation intensity 80%; excitation duration 0.8 s; gain 140; read interval 1/s; read number 300/addition. Responses mediated via endogenously expressed ion channels were elicited by addition of: choline (30 μM)/PNU-120596 (10 μM) for α7 nAChR-mediated responses; nicotine (30 μM) for α3β2/α3β4 nAChR-mediated responses; KCl (90 mM)/CaCl_2_ (5 mM) for Ca_V_1.3-mediated responses; KCl (90 mM)/CaCl_2_ (5 mM) + nifedipine (10 μM) for Ca_V_2.2-mediated responses; vertridine (70 μM) for Na_V_1.2-mediated responses; and veratridine (4 μM)/OD1 (300 nM) for Na_V_1.7-mediated responses. Raw fluorescence readings were converted to response over baseline using the analysis tool Screenworks 3.1.1.4 (Molecular Devices) and were expressed relative to the maximum increase in fluorescence of control responses and plotted using GraphPad Prism 9.3.1.

### Fiber mounting and force recording

All experiments were approved by The University of Queensland Animal Ethics Committees. Male and female C57/Bl6 2–6-month-old mice were sacrificed by asphyxiation via CO_2_ exposure and the EDL muscles were rapidly excised. The EDL was pinned at resting length in a petri dish lined with Sylgard 184 (Dow Corning, Midland, MI) and immersed in paraffin oil (Ajax Chemicals, Sydney, Australia). Segments of individual fibers were mechanically skinned using jeweler’s forceps and pinned out unstretched under oil^37–39^. The skinned fiber was then mounted at 120% of resting length on a force transducer (Myotronic UG, Heidelberg, Germany) before transfer to a 2-ml Perspex bath containing standard K^+^-based solution that broadly mimics the intracellular milieu. The standard solution for skinned fibers contained (in mM): hexa-methylene-diamine tetraacetate (HDTA^2−^), 50 (Fluka, Buchs, Switzerland); total ATP, 8; Na^+^, 36; K^+^, 126; total Mg^2+^, 8.5 (giving 1 mM free [Mg^2+^]); creatine phosphate, 10; total EGTA, 0.05; Hepes, 90; pH 7.1 and pCa (-log10[Ca^2+^]) ~7.1, except where stated. All chemicals were purchased from Sigma-Aldrich (St Louis, MO, USA) unless specified otherwise.

Variations of the standard solution were prepared. To deplete the caffeine releasable Ca^2+^ (“full release solution”), 30 mM caffeine was added, [Mg^2+^] was lowered to 0.05 mM (total Mg^2+^ of 2.1 mM) and free EGTA was adjusted to 0.5 mM (pCa 8.5) to chelate released Ca^2+^. To measure the maximum force produced by the skinned fiber, all HDTA was replaced by EGTA (relaxing solution) or CaEGTA (maximum Ca^2+^ activating solution). The relaxing solution contained 50 mM EGTA and no added Ca^2+^ (pCa > 9) and the maximum Ca^2+^-activating solution contained 49.5 mM Ca^2+^ (pCa 4.7), with total Mg^2+^ of 10.3 and 8.1 mM, respectively, to maintain the free [Mg^2+^] at 1 mM. A “leak solution” was also prepared, where [Mg^2+^] set at 0.1 mM (total Mg^2+^ of 3.4 mM) to increase the *P*_*O*_ of RyR and with 2 mM EGTA (pCa ~8.5) to chelate leaky Ca^2+^ from the SR. In addition, 10 nM of either Xt3a, Xt3a-D1, Xt3a-D2 or IpTxA, were added from their respective stock solution to the control leak solution to determine the individual effect of these peptides on the SR Ca^2+^ leakage. The BAPTA solution used to pre-equilibrate fibers before lysing was similar to the standard K-HDTA solution but EGTA was replaced with 0.1–1.6 mM BAPTA^37,39^.

### Quantifying total Ca^2+^ content in the fiber

Briefly, the skinned fiber was first placed in the standard weakly Ca^2+^-buffered K-HDTA solution for 2 min which does not alter the endogenous Ca^2+^ content of the fiber. The skinned fiber was then equilibrated for 20 s in a solution with a set [BAPTA] before being placed in a freshly triturated emulsion of Triton X-100 in paraffin oil (TX–oil) (10% v/v). The Ca^2+^ released upon the membrane lysing rapidly binds to the known amount of BAPTA present within the fiber and to other sites, predominantly troponin C (TnC). The pre-equilibrating [BAPTA] was chosen such that the fiber produced a finite, non-maximal force response upon lysis. Pre-treatment of the fiber with “full release solution” allowed the non-SR Ca^2+^ content of the skinned fiber to be assayed. The total amount of Ca^2+^ present in the fiber could be calculated from the [BAPTA] in the equilibration solution and the magnitude of the force response. Data are presented as mean ± SD, with *n* denoting the number of fibers examined. Statistical significance (*p* < 0.05) was determined with One-way ANOVA.

### Structure determination

The structure of Xt3a was determined using heteronuclear NMR. The sample contained 500 µM of ^13^C/^15^N-labeled Xt3a in 20 mM sodium phosphate pH 8, 10 µM DSS, 5% D_2_O, 1 mM EDTA and 0.02% NaN_3_. All spectra were recorded at 25 °C on a Bruker 900 MHz spectrometer equipped with a triple resonance cryogenically cooled probe (Bruker, Billerica, MA). Resonance assignments were obtained from two-dimensional (2D) ^1^H-^15^N-HSQC, 2D ^1^H-^13^C-HSQC, 3D HNCACB, 3D CBCA(CO)NH, 3D HNCO, and 3D HBHA(CO)NH, 3D H(CCCO)NH, 3D (H)C(CCO)NH and 3D HCCH-TOCSY (BMRB ID: 30944). The 3D spectra were acquired using nonuniform sampling and processed using maximum entropy reconstruction with the Rowland NMR Toolkit as described previously^57^. The interproton distance restraints used for structure calculations were derived from 3D ^1^H-^13^C-aliphatic, ^1^H-^13^C-aromatic, and ^1^H-^15^N NOESY spectra acquired using uniform sampling with a mixing time of 150 ms. The spectra were analysed using CcpNmr v2.4.1^58^. Dihedral-angle restraints were derived from TALOS-N chemical shift analysis program^59^, with the restraint range set to twice the estimated standard deviations. The NOESY spectra were manually peak-picked and the torsion angle dynamic package CYANA v3.98.13^60^ was then employed to automatically assign the peaks, extract distance restraints and calculate an ensemble of structures. The disulfide-bond connectivity was determined from preliminary structure calculations performed without any disulfide-bond restraints^61^. The inter-cystine NOEs in the NOESY spectra are consistent with the pattern Cys14-Cys29, Cys21-Cys34, Cys28-Cys45, Cys50-Cys65, Cys57-Cys70 and Cys64-Cys80. CYANA calculated 200 structures from random starting conformations and the best 10 conformers were selected, based on their final CYANA target function value, to represent the structure of Xt3a. Coordinates for the Xt3a ensemble are available from the Protein Data Bank (PDB ID: 7RZ3).

### Production and circularisation of membrane scaffold protein MSP9

The method for producing circularised MSP9 (cNW9) was as described in Nasr et al.^62^. In brief, the MSP9 gene containing a sortase A (SrtA) recognition sequence (LPGT) in the C-terminal was cloned into pET29a vector and the protein was produced recombinantly in *E. coli* BL21 (DE3). After purification using IMAC, the MSP9 was circularised using SrtA in reaction buffer containing 25 mM Tris·HCl pH 7.5, 150 mM NaCl, 0.5 mM EDTA, 2 mM DDM and 1 mM 2-mercaptoethanol (BME). The molar equivalent ratio of MSP9:SrtA in the reaction was 1:1 and the reaction was initiated by the addition of 10 mM CaCl_2_. The reaction was carried out at 37 °C for 16 h.

Following the completion of circularisation, the sample was purified by reverse-IMAC to remove any unreacted material and SrtA. In this step, the Ni-NTA resin was pre-equilibrated with buffer containing 25 mM Tris·HCl pH 7.5, 150 mM NaCl and the flow-through containing cNW9 was collected. The flow-through fraction was then desalted into equilibration buffer (25 mM Tris·HCl pH 7.5, 1 mM DDM) and it was applied onto a 5 mL HiScreen Q HP (GE Healthcare) anion-exchange column at 4 °C using an ÄKTA FPLC system (GE Healthcare). At a flow rate of 0.3 mL/min, a linear gradient from 0 to 30% of equilibration buffer supplemented with 1 M NaCl over 20 column-volumes (CVs) was then applied to elute the pure cNW9. The purity of the fractions was assessed by SDS-PAGE and fractions containing cNW9 with >95% purity were pooled for nanodisc (ND) assembly.

### POPC-cNW9 nanodisc assembly

The method of circularised ND (cND) assembly was developed based on previous reports^62,63^. A stock solution containing 20 mg of POPC in chloroform was dried down under nitrogen gas in a glass tube and the lipid film was left under vacuum overnight. The dried film was rehydrated with reconstitution buffer (20 mM Tris·HCl pH 7.4, 100 mM NaCl, 0.5 mM EDTA and 200 mM cholate) to make POPC stock at 100 mM. The solution was sonicated for 10 mins in a warm water bath at 60 °C to fully reconstitute the lipids in the buffer. To assemble the cND, cNW9 solution and POPC/cholate stock were mixed at a cNW9:POPC:cholate molar ratio of 1:43:86. The mixture was incubated for 1 h on ice with gentle rocking and the assembly was initiated by the addition of 0.5–1 g of Bio-Beads SM-2 (Bio-rad) to every mL of reaction volume. The mixture was kept on ice with rocking for another 3 hours for the Bio-Beads to remove the detergent in the sample. The solution was filtered through a 0.45 μm PES membrane to remove the Bio-Beads and then directly injected onto a Superdex 200 increase 10/300 column (GE Healthcare) equilibrated with 20 mM Tris·HCl pH 7.4, 100 mM NaCl and 0.5 mM EDTA using a ÄKTA FPLC system (GE Healthcare). Fractions containing POPC-cNW9 ND were pooled and the homogeneity and size distribution of the cND sample was assessed using negative-stain EM.

### Negative Stain TEM

400-mesh carbon-coated (5–6 nm thick) copper grids (ProSciTech Pty Ltd, Kirwan, Australia) were glow-discharged for 15 s before the protein was applied. 4 µL of POPC-cNW9 ND at 20 nM (20 mM Tris·HCl pH 7.4, 100 mM NaCl and 0.5 mM EDTA) was pipetted onto the grid and allowed to settle for 1 min. This was followed by one wash step in a drop of water and the grid was immediately moved to a fresh drop of 1% uranyl acetate to stain for 1 min. TEM was carried out on a Hitachi HT7700 electron microscope at 80 kV.

### NMR titrations of POPC-cNW9 nanodiscs to Xt3a

^1^H-^15^N-HSQC spectra of Xt3a with and without the presence of ND were recorded on a 900 MHz spectrometer equipped with a cryogenically cooled triple resonance probe (Bruker, Billerica, MA) at 25 °C. Each sample contained 50 μM of ^15^N/^13^C-labelled Xt3a in 20 mM sodium phosphate pH 8.0, 10 µM DSS, 5% D_2_O, and 1 mM EDTA, either with no or 100 μM POPC-cNW9 ND. Spectra were processed and analysed using Topspin (v4.1.3, Bruker).

### Relaxation experiments

^15^N spin-lattice (R_1_), transverse (R_2_) relaxation, and ^1^H-^15^N NOE experiments for Xt3a were recorded at 25 °C on a 900 MHz spectrometer equipped with cryogenic probe (Bruker, Billerica, MA). The sample contained 500 μM of ^15^N/^13^C labeled Xt3a (as above). The relaxation delay was sampled at 10, 20, 60, 100, 200, 400, 600 and 1200 ms for the R_1_ experiments and 16, 33, 67, 135, 169, 203, 237 and 271 ms for the R_2_ measurements. Spectra were processed using Topspin (v4.1.3, Bruker) and the signal decay was analysed and plotted using CcpNmr (v2.4.1). The time constant error (TC in CcpNmr) from the exponential fit is used in the the R_1_ and the R_2_ plots. The noise in the spectrum (relative to the peak height) was used to estimate the uncertainty (error bars) in the heteronuclear NOE plots.

### Evolutionary analyses

To explore the evolutionary origin of Xt3a, we used blastp (blast+ v.2.10.1 + )^64^ to search both the full-length and single domains against a database of all 228,896 mRNA sequences listed under Remipedia in NCBI and translated to all potential open reading frames encoding 40 amino acids or more (880,562 sequences). We also searched the trinity-assembled venom gland and whole-body transcriptomes reported by von Reumont et al.^17^, the arthropod component of a custom database containing trinity-assembled transcriptomic datasets from 125 species of arthropods obtained from the NCBI SRA reported previously^65,66^, as well as the NCBI nr and UniProtKB databases. We used standard blastp settings and retained all sequence hits containing at least one putative ICK domain, identified as containing either of the canonical cysteine patterns corresponding to either of the three- or four-disulfide ICK forms (3-DS and 4-DS, respectively). Given that we did not identify any single-domain 4-DS among the remipede sequences using blastp, we also queried the remipede databases with jackhmmer (HMMER v3.1b2^67^) to iteratively search all remipedes 3-DS and 4-DS ICKs against the same protein databases, using an inclusion threshold score of 30 and once again manually filtering out non-ICK sequences.

The identified sequences were combined to a single fasta file and clustered sequences with a similarity greater than 99% using CD-HIT v4.6.5^68^. Of the pancrustacean single-domain ICKs, GSCOCT00013103001.2-RA-CDS_S6D9L2 from *Cotesia congregata* was removed due to very long inter-cysteine loops and because its insect clade (Hymenoptera) is relatively well represented, X1X2U9 from *Acyrthosiphon pisum* was removed due to an unusual propeptide or very long N-terminus which could be due to frameshift, while the remipedes sequence JL112461.1 and A0A0B4UDE1 from *Drosophila mojavensis* were removed due to missing cysteines. We then split each sequence containing multiple ICK domains into separate sequences for each domain and aligned all ICK domains using the local paired iterative alignment method (L-INS-i) in MAFFT v7.304b64^69^. We then realigned the structurally important conserved cysteines^5^, and used the MAFFT regional alignment ruby script to align the pre-, inter-, and post-cysteine regions by local paired iterative alignment as above. We selected the most appropriate evolutionary model (VT + I + G4) using ModelFinder^70^, before we used IQ-TREE v1.5.5^71^ to reconstruct the molecular phylogeny of our ICK domains by maximum likelihood, estimating branch support values by ultrafast bootstrap using 10,000 replicates^72^. Trees were visualised in Archaeopteryx v0.9921^73^. We also used IQ-TREE to estimate the site-specific substitution rates by maximum likelihood of the remipede double-ICK peptides, which were aligned using MAFFT as above.

To examine the convergent evolution of Xt3a and toxins belonging to the calcin family, we downloaded all functionally annotated ICK venom peptides from UniProtKB, trimmed them to contain only mature peptide domains, removed identical sequences using CD-HIT, and aligned these and the individual domains of all remipede ICK peptides using the regional alignment approach described above. We then clustered and projected the biophysical properties of each sequence in a multidimensional sequence space as described previously^66,74^, using [R] codes obtained from https://github.com/TS404/SeqSpace. Briefly, the alignment was converted into a numerical matrix and analyzed using principal component analysis in R to summarize the main covarying sets of properties. The variables used were molecular weight (Da), net charge (Coulombs), hydrophobicity (Doolittle index), disorder propensity (TOP-IDP), disulfide potential (binary descriptor) and occupancy (binary descriptor).

### Statistical analysis

In the cell-based FLIPR assays statistical analysis was performed using GraphPad Prism 9.3.1. Statistical significance was defined as *p* < 0.05, and determined by one-sample *t*-test assessing difference to the control value of 100%. In α7 nAChR, α3-containing nAChR, Ca_V_1.3, Ca_V_2.2, Na_V_1.2 and Na_V_1.7 channels (Supplementary Fig. 5B), with distribution (t) and degrees of freedom (df) being: *t* = 0.9683, df = 2 (α7); *t* = 1.338, df = 2 (α3); *t* = 1.775, df = 2 (Na_V_1.2); *t* = 1.478

Due to the well-recognized range of values between individual RyR channels, in the electrophysiology experiment, all parameters (*P*_*o*_, *T*_*o*_*, T*_*c*_ and *F*_*o*_) for each channel are expressed relative to the internal control values for that channel, measured prior to application of Xt3a. Occasional measurements were excluded from data sets when with *P*_*O*_ values were 10 to 30 times outside the range of other channels in the same experiment. This included 1 of 16 measurements in 8 RyR1 channels exposed to 10 to 100 pM Xt3a; 1 of 16 measurements in 8 RyR1 channels exposed 1 nM and 10 nM Xt3a; 4 of 16 measurements from 8 RyR2 channels exposed to 1 and 10 nM Xt3a, and 3 of 18 measurements from 9 RyR2 channels exposed to 0.1 and 1 μM Xt3a. Statistical analysis of single channel data was performed using Excel spreadsheets and embedded Students *t*-test functions or a sign test as indicated in the graphs in Figs. 1 and 3. For the *t*-test, a 2-tailed paired *t*-test was used for the data sets (*n* = 6 to 10 channels) shown in Fig. 1b and c and for the subset of RyR2 channels maintained through Xt3a washout shown in Fig. 3b (*n* = 5). A 1-tailed paired *t*-test was used with the subset of RyR1 maintained through Xt3a washout shown in Fig. 3a (*n* = 4), as significant increases in activity had been demonstrated in the larger data set shown in Fig. 1.

### Reporting summary

Further information on research design is available in the Nature Portfolio Reporting Summary linked to this article.

## Supplementary information

Supplementary Information

Description of Additional Supplementary Files

Supplementary Data 1

Supplementary Data 2

Reporting Summary

## Data Availability

The data that support this study are available from the corresponding authors upon reasonable request. Coordinates have been deposited in the Protein Data Bank (PDB) under accession code 7RZ3 (Xt3a) and related NMR data deposited in the Biological Magnetic Resonance Data Bank under accession code 30944 (The solution structure of remipede double-ICK toxin phi-Xibalbin3-Xt3a). Source Data underlying figures are included as a Source Data file. Source data are provided with this paper.

## Source data

Source Data

## Code availability

Processing scripts and code available from the authors upon request.
