# Supplementary material for: A bivalent remipede toxin promotes calcium release via ryanodine receptor activation
Source: Nat Commun. 2023 Feb 23;14:1036. doi: 10.1038/s41467-023-36579-w (PMC9950431; doi:10.1038/s41467-023-36579-w)
Supplement: Supplementary file 1 — Supplementary Information [file 41467_2023_36579_MOESM1_ESM.pdf]

Supplementary Information for

A bivalent remipede toxin promotes calcium release via ryanodine receptor activation

Michael J. Maxwell[1a], Chris Thekkedam[2], Cedric Lamboley[1b], Yanni K. –Y. Chin[1a], Theo Crawford[1a], Jennifer J. Smith[1c], Junyu Liu[1a], Xinying Jia[1a], Irina Vetter[1c], Derek R. Laver[2], Bradley S. Launikonis[1b], Angela Dulhunty[3], Eivind A. B. Undheim[1a,c,4*], Mehdi Mobli[1a*]

Affiliations

[1a]Centre for Advanced Imaging, [b]School of Biomedical Sciences, [c]Institute for Molecular Bioscience, The University of Queensland, St. Lucia, QLD 4072, Australia.

[2]School of Biomedical Sciences and Pharmacy, University of Newcastle, Newcastle 2308, Australia

[3]Eccles Institute of Neuroscience, John Curtin School of Medical Research, Australian National University, Canberra, ACT 2601, Australia

[4]Centre for Ecological and Evolutionary Synthesis, Department of Biosciences, University of Oslo, 0316 Oslo, Norway

*Corresponding Authors: Eivind A. B. Undheim, e.a.b.undheim@ibv.uio.no or Mehdi Mobli, m.mobli@uq.edu.au

This PDF file includes:

Supplementary Figs. 1 to 14
Supplementary Tables 1 to 3
Supplementary Data 1 to 2

Supplementary Figures

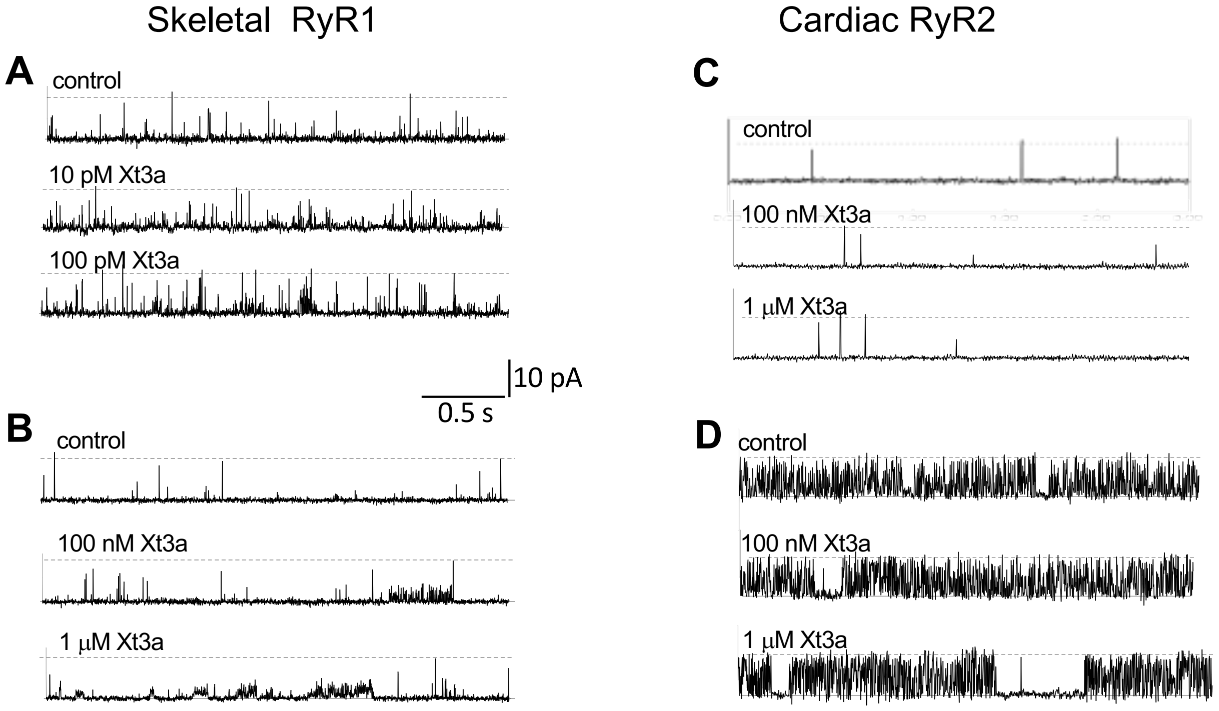

Supplementary Fig. 1.

Xt3a activates RyR1 at picomolar concentrations and inhibits channels at concentrations between 100 nM and 1 µM, but has no general effect on cardiac RyR2 channels. (A-B) shows traces from two different RyR1 channels at +40 mV exposed to 10 pM and 100 pM Xt3a (A), or 100 nM and 1 µM Xt3a (B). Note the increasing RyR1 channel opening to sub maximal conductance levels as [Xt3a] increases. (C-D) shows recordings from two different RyR2 channels exposed to 100 nM and 1 µM Xt3a, one with low endogenous activity (C), and the other with high endogenous activity (D).

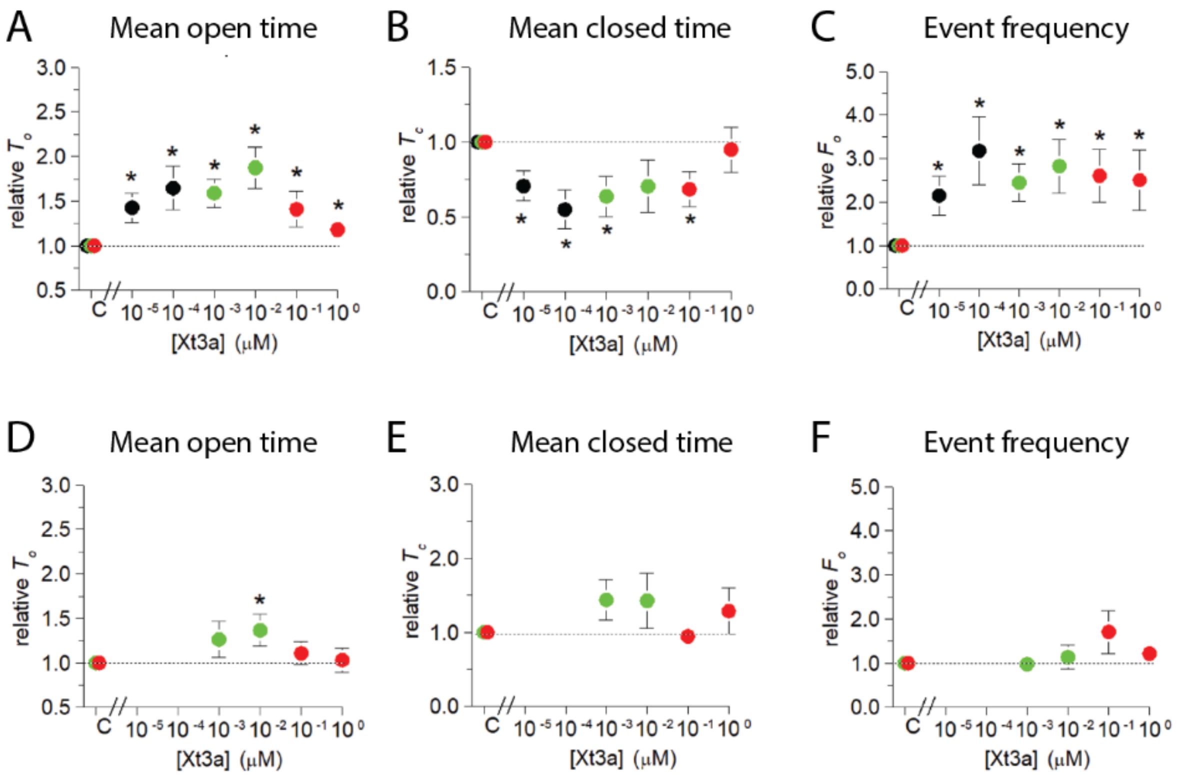

Supplementary Fig. 2.

Xt3a induces significant changes in gating parameters in RyR1 channels, but not in RyR2. (A-F) Average normalized mean open time (T_o), relative mean closed time (T_c) and relative open event frequency (F_o) are plotted against [Xt3a] for RyR1 (A to C) and RyR2 (D to F). Data are colour coded to indicate data obtained at 10 pM and 100 pM (black: RyR1, n=7: T_o, P=2.07E-02, P=2.02E-02; T_c, P=1.03E-02, P=4.02E-03; F_o, P=1.53E-02, P=1.49E-02), 1 nM and 10 nM (green: RyR1 n=7: T_o, P=2.12E-03, P=2.42E-03; T_c, P=1.60E-02, P=1.25E-01; F_o P=4.42E-03, P=9.92E-03. RyR2, n=6: T_o, P=2.24E-01, P=3.21E-02; T_c, P=1.42E-01, P=2.83E-01; F_o P=9.14E-01, P=6.29E-01) and 100 nM and 1 μM (red: RyR1 n=10 T_o, P=2.65E-02, P=2.84E-02; T_c, P=1.45E-02, P=3.71E-01; F_o, P=1.24E-02, P=4.28E-02; RyR2 n=6: T_o, P=4.22E-01, P=8.16E-01; T_c, P=6.83E-01, P=3.76E-01; F_o P=1.74E-01, P=2.67E-01. Asterisks indicate values that are significantly different from control. The symbols show mean±SEM and n refers to the number of observations included in the mean. Two independent observations obtained at +40 mV and -40 mV, measure current flow in opposite directions through the channel pore, are included as the effect of Xt3a was similar at the two potentials. Significance was determined using a two sided Students t-test.

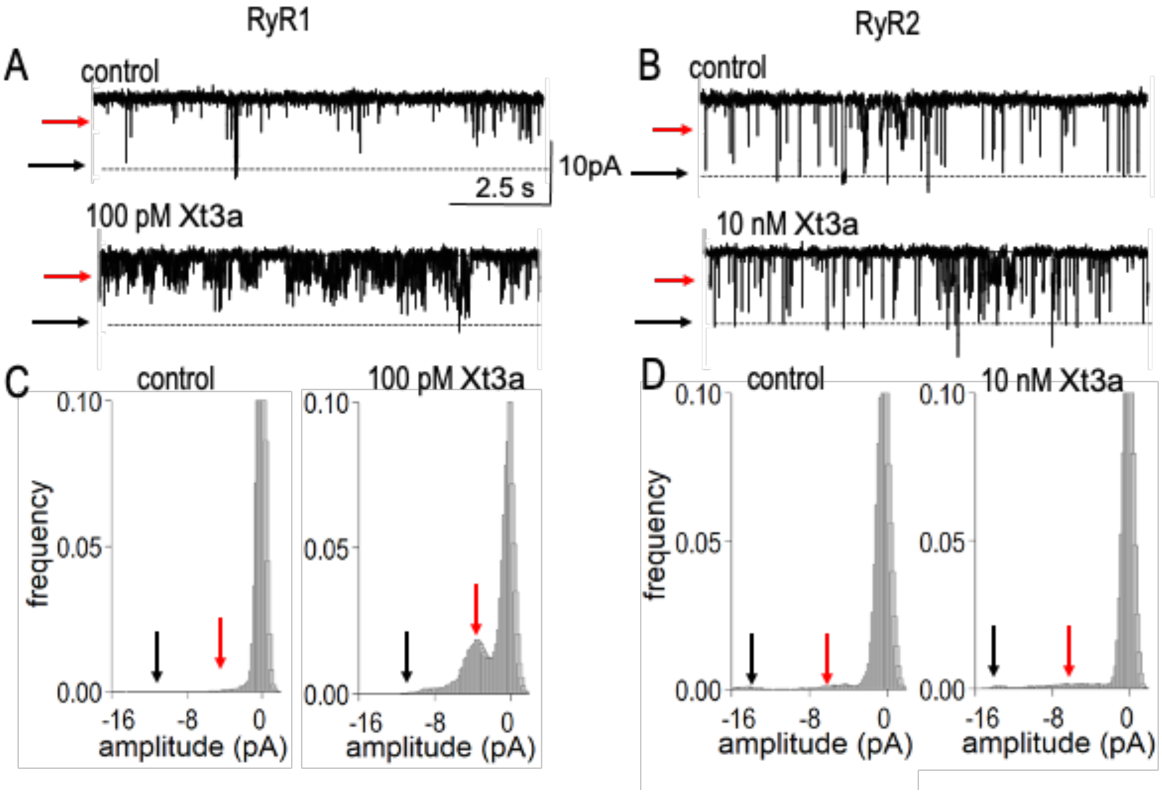

Supplementary Fig. 3.

Strong stabilization of substate activity by Xt3A in RyR1 at -40 mV. **A**) and **B**) show ten second recordings from RyR1 and RyR2 channels at -40 mV with Xt3a: in **A**) from an RyR1 channel before exposure (control) and after exposure to 100 pM Xt3a and **B**) from an RyR2 channel, control and with 10 nM Xt3a. The recordings demonstrate prolonged periods of channel gating to substate levels at <50% of the maximum conductance. The broken line in each of the recordings marks the maximum conductance level. **C**) and **D)** show amplitude histograms for RyR1 (**C**) and RyR2 (**D**) from the recordings shown above. In **A**) to **D**) the black arrows indicate the maximum open current level and the red arrows point to the predominant substate current level.

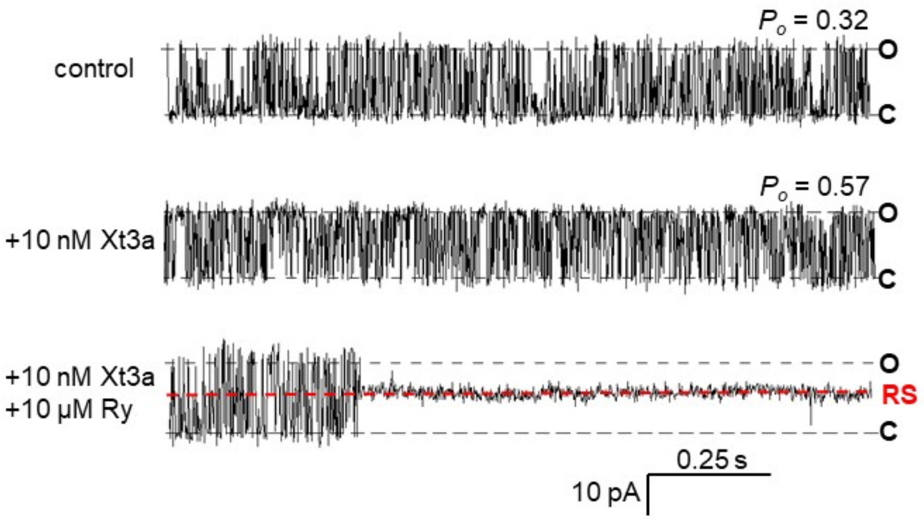

Supplementary Fig. 4.

Electrophysiological response after addition of Xt3a and ryanodine. Recordings from RyR1 +40 mV from one experiment representative of four. A single high activity channel was obtained under control conditions (control), and after 1 min exposure to 10 nM Xt3a (Xt3a) and after 4 s of further addition of 10 µM ryanodine (Ry). The classical ryanodine block of normal channel gating and entry into the substate at ~50% of the fully open conductance commences appoximately 0.35 s into the trace.The parallel lines (labelled C and O) indicate the closed (**C**) level and fully open (**O**) levels repectively. The red line (labelled **RS**) indicates the ryanodine substate. Open probability (P_o) is shown above the control and Xt3a records.

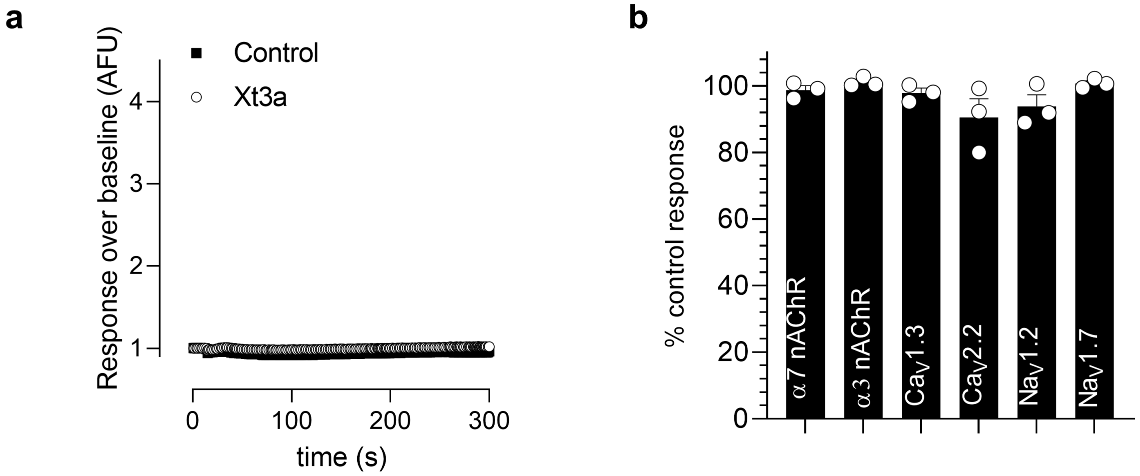

Supplementary Fig. 5.

Xt3a does not elicit Ca^{2+} responses or inhibit endogenously expressed ion channels in SH-SY5Y cells. (a) Addition of Xt3a (3 µM) does not elicit an increase in intracellular Ca^{2+} responses. (b) Xt3a (3 µM) does not inhibit endogenously expressed α7 nAChR, α3-containing nAChR, $Ca_V1.3$, $Ca_V2.2$, $Na_V1.2$ and $Na_V1.7$ channels. Responses over 300 s after pre-treatment with Xt3a (3 µM) were expressed relative to control responses elicited by subtype-selective agonists. Experiments were performed in triplicate, data is presented as mean ± standard deviation.

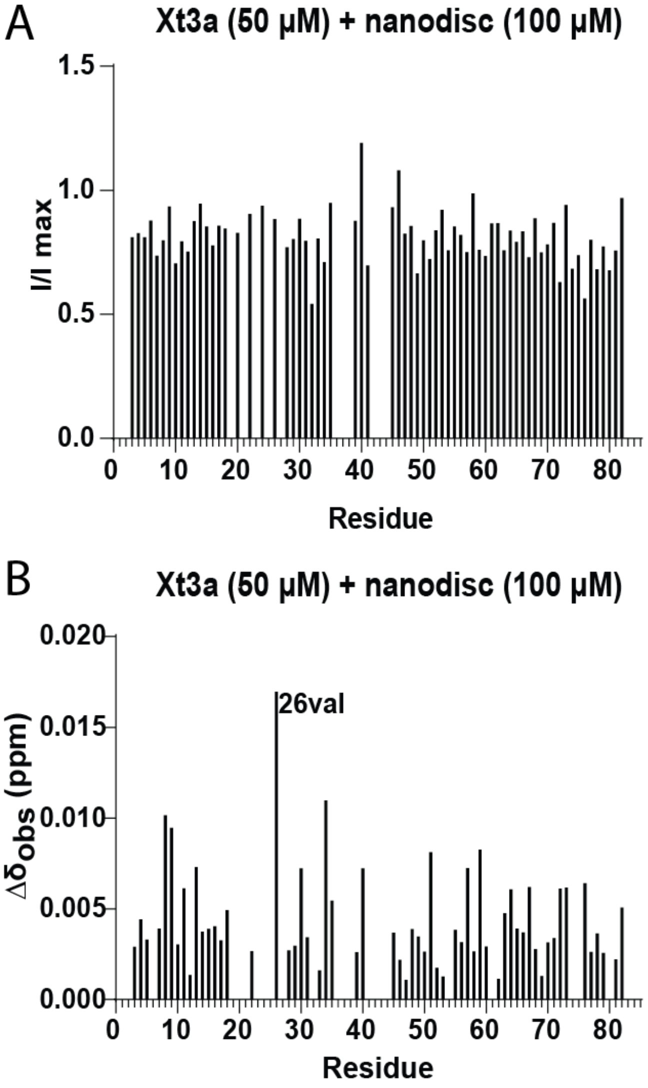

Supplementary Fig. 6.

Xt3a binds weakly and non-specifically to lipid bilayers. ^{15}N-HSQC measurements of Xt3a with and without nanodiscs containing zwitterionic (POPC) lipids. (A) Changes in peak intensity for each amide resonance (I/I_{max}). (B) Observed chemical shift differences for each amide resonance ($\Delta\delta_{obs}$ (ppm)).

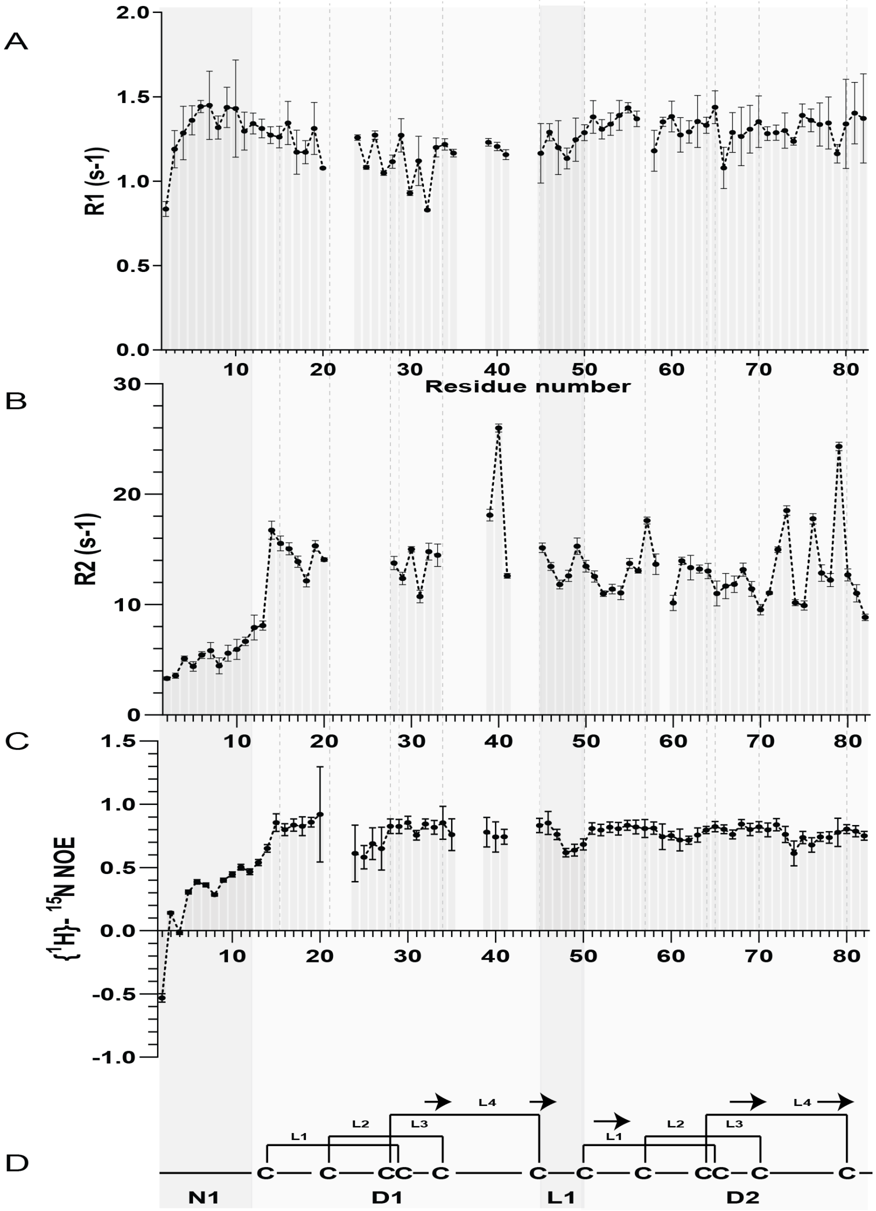

Supplementary Fig. 7.

Dynamics of Xt3a from backbone ^{15}N-NMR spin relaxation experiments. (A) ^{15}N-R1 relaxation rate values derived from fitting to measurements for each backbone amide. Residue Cys57 was removed due to poor signal quality (error bars based on fitting uncertainty of the time constant). (B) ^{15}N-R2 relaxation rate values derived from fitting to measurements for each backbone amide. Residue Leu59 was removed due to poor signal quality (error bars based on fitting uncertainty of the time constant). (C) ^{1}H-^{15}N heteronuclear NOE ratios (errors based on spectral noise). No amide resonances were observed between residues positions 21-23, 36-38, and 42-44. (D) Schematic of Xt3a architecture, highlighting the N-terminal region (N1), each disulfide rich domain (D1, D2), and the -connecting linker region L1. The cysteine connectivity is shown with horizontal brackets, the inter-cysteine loops labelled L1-L4 with dashed lines highlighting their corresponding position in the above plots, and arrows indicating the anti-parallel β-sheets.

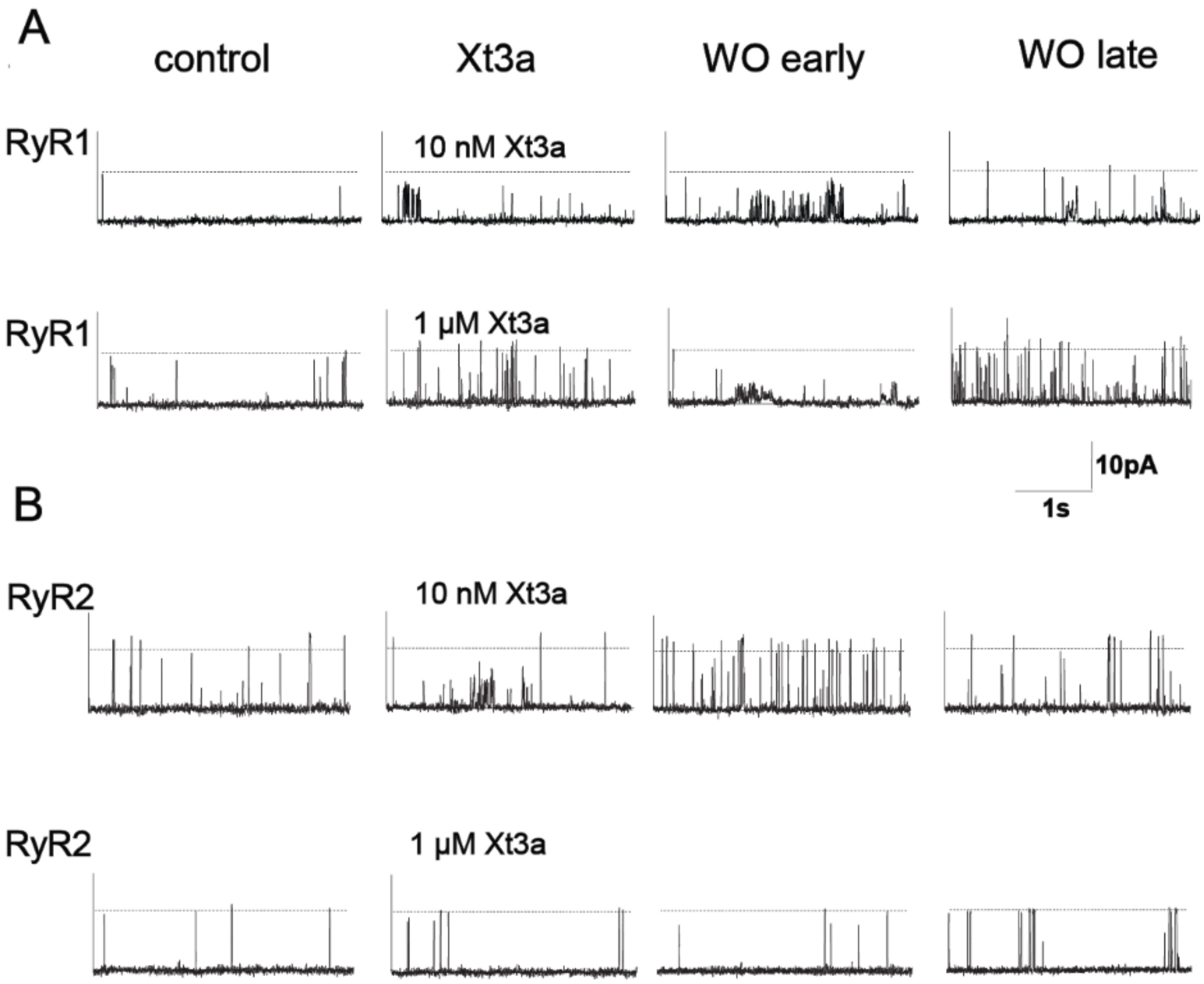

Supplementary Fig. 8.

Limited reversibility of Xt3a effects on RyR1 channels, consistent with the apparent high affinity binding of the toxin to the channel. The records shown contribute to the average data in Figure 3 of the manuscript. Details of quantification and statistics are given in the manuscript for the subset of data given in the Figure 3. Recordings from RyR1 channels are shown in (A) and from RyR2 channels are shown in (B). In (A) and (B), recordings from representative channels at +40 mV are shown, under control conditions (column 1), after ~10 min exposure to the indicated concentration of Xt3a (column 2), within 3 min removal of Xt3a by perfusion of the cis chamber (column 3) and finally ~15-20 min after perfusion (column 4).

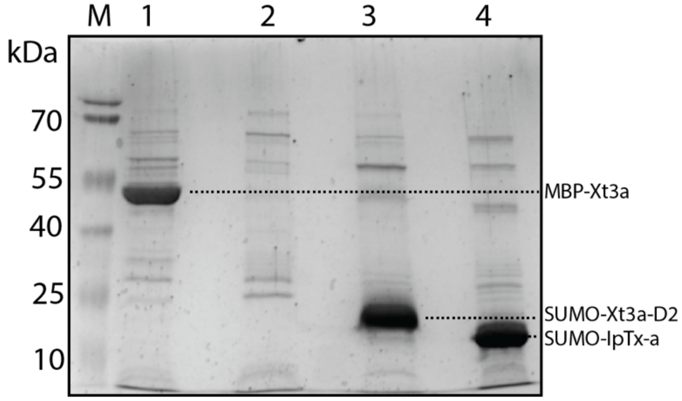

Supplementary Fig. 9.

SDS-PAGE representative of the purification of MBP and SUMO fusion protein constructs. Each lane corresponds to: M, molecular mass markers; 1, eluate of the MBP-Xt3a fusion protein from the Ni-NTA beads; 2, eluate of SUMO-Xt3a-D1 (unsuccessful purification); eluate of SUMO-Xt3a-D2 fusion protein from Ni-NTA beads; 4, and eluate of SUMO-IpTxA fusion protein from Ni-NTA beads.

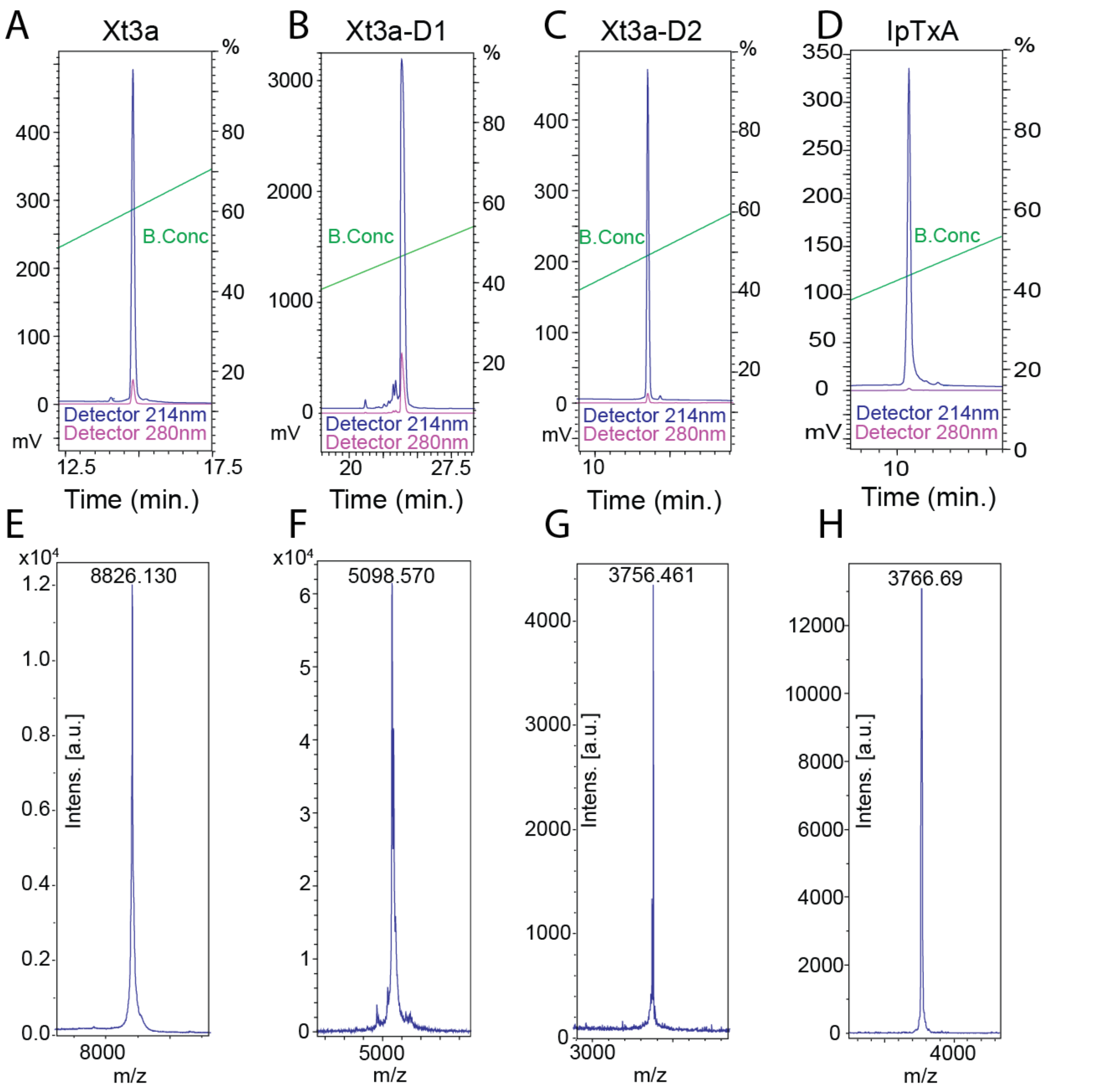

Supplementary Fig. 10.

Purification and mass analysis of recombinantly produced Xt3a, Xt3a-D1, Xt3a-D2, and IpTxA. (A, B, C, D) Analytical RP-HPLC chromatogram showing pure recombinant Xt3a, Xt3a-D1, Xt3a-D2, and IpTxA. (E, F, G, H) Matrix-assisted laser desorption/ionization (MALDI-TOF) spectrum of purified Xt3a, Xt3a-D1, Xt3a-D2, and IpTxA.

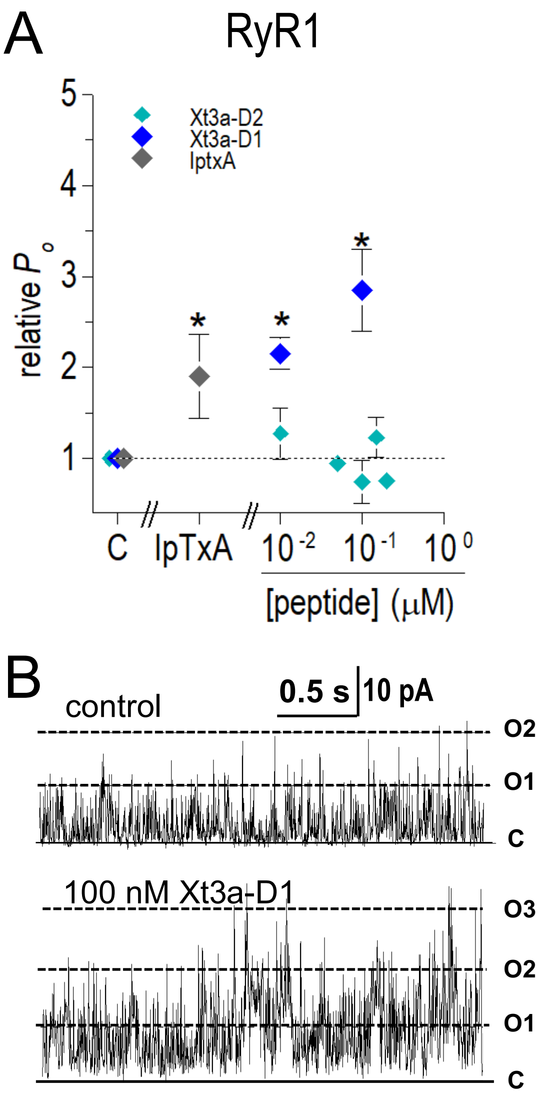

Supplementary Fig. 11.

Xt3a and Xt3a-D1 activate ryanodine receptors (RyRs) in a similar way to other toxins in the calcin family. (A) data color coded to differentiate peptides examined. Blue: Xt3a-D1, 10 nM, n=6, P=1.31E-03; 100 nM, n=7, P=3.23E-03; Cyan: Xt3a-D2, 10 nM, n=6, P= 3.07E-01; 50 nM, n=3, 6.93E-01; 100 nM n=3, P=3.93E-01; 150 nM, n=3, P=4.00E-01; 200 nM, n=3, P=1.56E-01. Charcoal: IpTxA concentrations tested (0.1 nM n=2, 1 nM n=3, 10 nM n=2) were grouped (n=7, P=3.7E-02) and show activation withn a similar range, and to previous reports[1]. Asterisks indicate values that are significantly different from control. The symbols show mean±SEM and n refers to the number of observations included in the mean. All data in this figure was obtained at +40mV. Significance of was determined using a two sided Students t-test. (B) shows 3 s from a RyR1 channel before and after exposure to 100 nM Xt3a-D1. The parallel lines (labelled C, O1, O2 and O3 in (G) indicate the closed level and open levels when 1, 2 or 3 channels respectively open simultaneously.

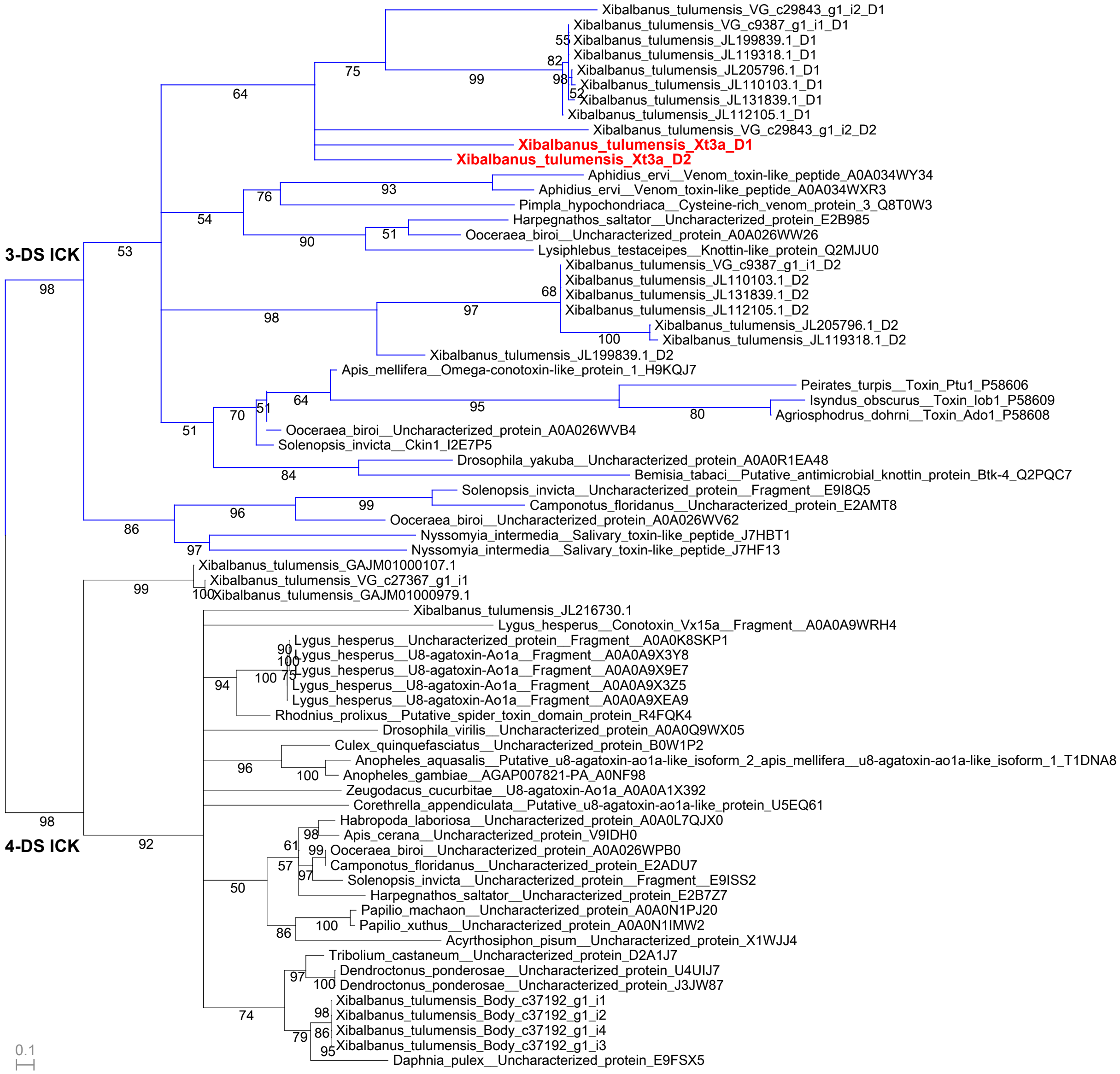

Supplementary Fig. 12.

Full maximum likelihood consensus tree generated under the evolutionary model VT+I+G4. Branch support values were estimated by ultrafast bootstrap using 10,000 replicates and branches with confidence < 50 are collapsed into multifurcations. Branches in the three-disulfide ICK clade (3-DS ICK) are coloured blue, while branches in the four-disulfide ICK clade (4-DS ICK) are coloured black. The two Xt3a domains are highlighted in bold red.

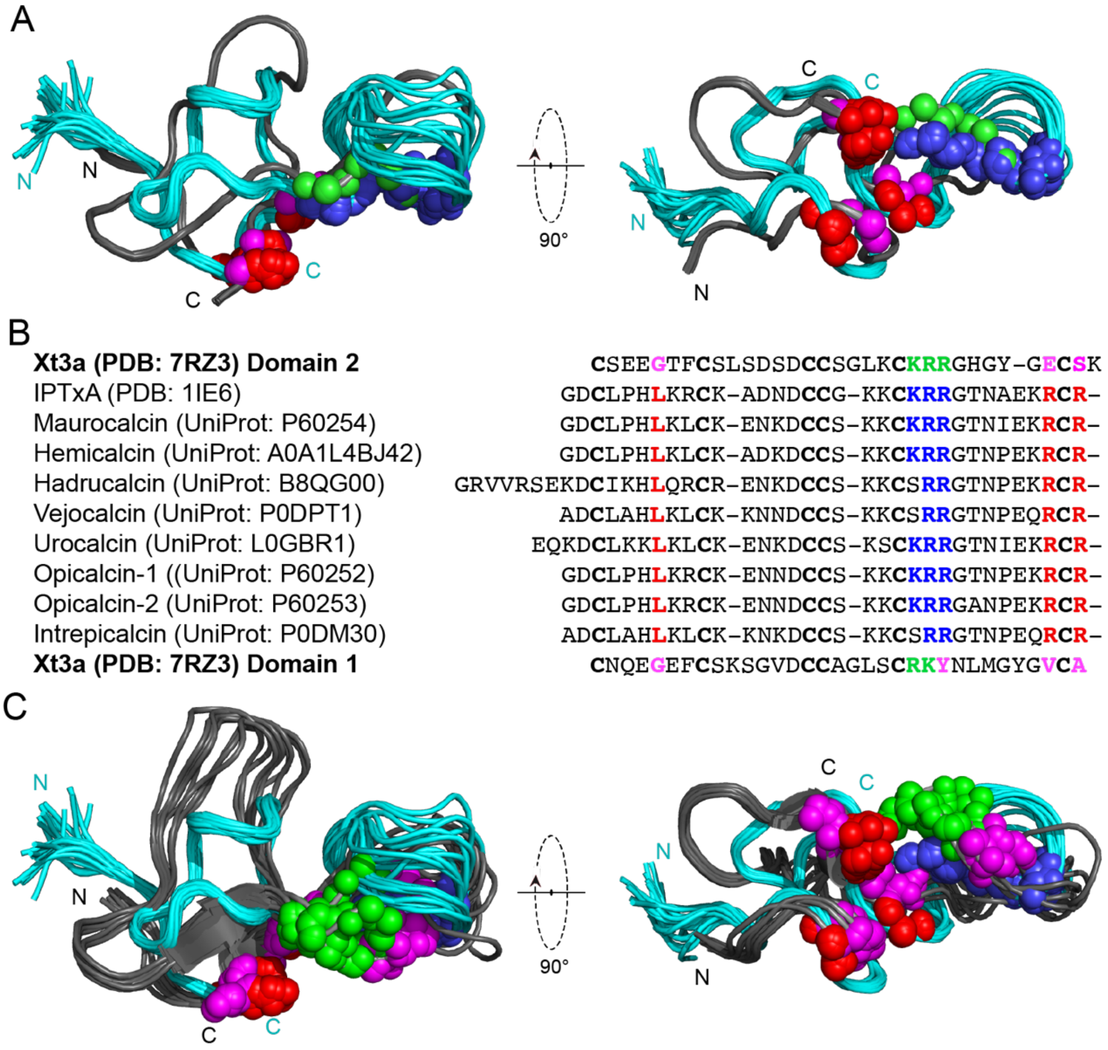

Supplementary Fig. 13.

Xt3a lacks many of the residues that are critical for RyR activity of calcins. (A) Xt3a-D2 lacks half of the structural interface thought to facilitate the binding of calcins to RyR. IpTxA (PDB 1IE6; cyan) aligns to Xt3a-D2 (dark grey) with an RMSD of 3.319 Å (pymol cealign). Locations of residues critical for RyR activity of IpTxA are indicated by spheres, where blue residues are present in both IpTxA and Xt3a-D2, while red residues are present in IpTxA only. The locations of the corresponding residues in Xt3a-D2 are indicated by green and magenta spheres, respectively. (B) Multiple sequence alignment of calcins against Xt3a-D2 (top) and Xt3a-D1 (bottom) with residues critical for RyR activity of calcins, and the corresponding residues in Xt3a-D2 and D1, highlighted in bold and coloured as in panel A. Cysteines are shown in bold but coloured black. (C) Structural alignment of Xt3a-D1(dark grey) to IpTxA (cyan; pymol cealign, RMSD 4.170 Å) showing that Xt3a-D1 lacks most of the structural interface thought to facilitate the binding of calcins to RyR. Residues are coloured as in panel A.

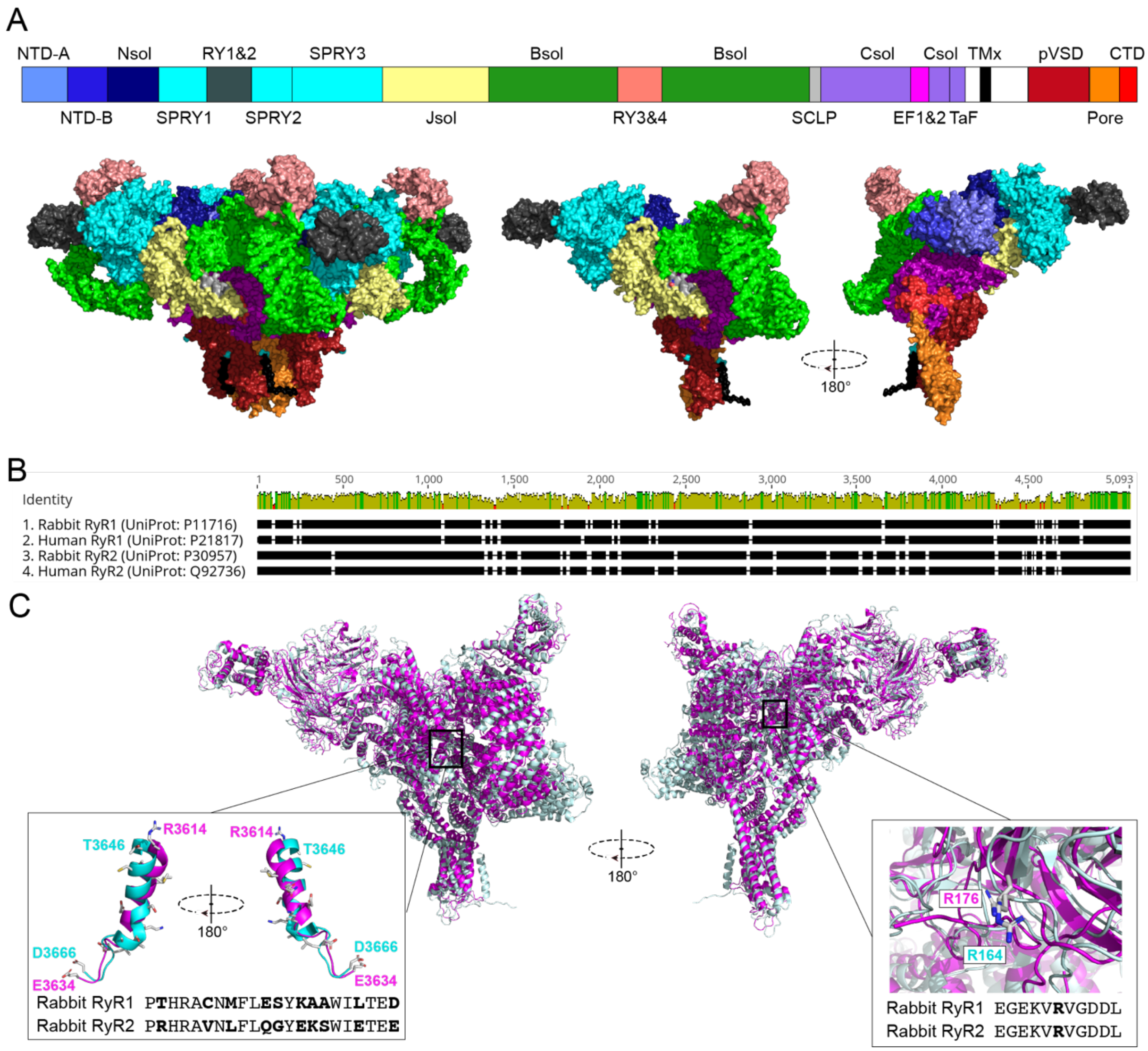

Supplementary Fig. 14.
Some structural differences between RyR1 and RyR2. (A) Top: Overview of the domain organisation of RyR. Bottom: Surface view of full (left) and single subunit (right) of rabbit RyR1 (PDB 5TB2) with domains coloured as the diagram above. Domain terminologies and boundaries are from Georges *et al.*[2]: N-terminal domain A (NTD-A); N-terminal domain B (NTD-B); N-terminal solenoid (Nsol); SP1a/ryanodine receptor domains 1-3 (SPRY1, SPRY2, SPRY3); RYR repeats 1&2 and 3&4 (RY1&2, RY3&4); junctional solenoid (Jsol), bridging solenoid (Bsol), shell-core linker peptide, CaM, and JSol binding sites (SCLP); C-terminal solenoid (Csol); EF-hand pair (EF1&2); auxiliary transmembrane helices (TMx); pseudo voltage sensor domain (pVSD); C-terminal domain (CTD). (B) Global view of multiple sequence alignment of human and rabbit RyR1 and RyR2 generated using the local paired iterative alignment method (L-INS-i) in MAFFT v7.304b64[3] and visualized using Geneious v2022.1 (Biomatters, NZ). Bars represent site-specific sequence conservation, where the majority of differences are between the two RyR paralogue pairs: while the RyR orthologues are overall much more conserved (96.532 % and 98.591 % pairwise sequence identity for RyR1 and RyR2, respectively) than the RyR paralogues (65.068 % and 65.107 % pairwise sequence identity for human and mouse, respectively). (C)

Structures of single subunits of rabbit RyR1 (PDB 5TB2; light cyan) and RyR2 (PDB 5L1D; magenta) from closed channels align with an RMSD of 9.097 Å across all aligned atoms although several outliers are visible, such as between the Bsol domains. Differences in primary structures of RyR1 and RyR2 are also present in well-aligned domains (e.g., C-terminal part of the SCLP domains; left inset; differing residues highlighted in bold and their side-chains shown as tubes). In addition, mutations of conserved, well-aligned residues may have completely different effects, such as the mutation of R164 in RyR1 and R176 in RyR2 (right inset; highlighted in bold and their side-chains shown as tubes) to a non-charged residue, which results in a much greater increase in open state probability in RyR1 than in RyR2[4].

Supplementary tables

Supplementary Table 1.

Structural statistics for the NMR ensemble of Xt3a[a]

PDB ID	7RZ3
Experimental restraints	
Inter-proton distance restraints	
Total	1326
Intra-residue ($i = j$)	330
Sequential ($\|i - j\| = 1$)	407
Medium range ($1 < \|i - j\| < 5$)	191
Long range ($\|i - j\| \geq 5$)	398
Disulfide bond restraints	18
Dihedral-angle restraints (ϕ, ψ)	146
ϕ dihedral angle restraints	67
ψ dihedral angle restraints	63
χ_1 angle restraints	16
Mean number of restraints per residue	18.2
Violations of experimental restraints	2
RMSD to mean coordinate structure (Å)	
All backbone atoms	2.30 ± 0.86
All heavy atoms	2.65 ± 0.87
Backbone atoms (residues 14-34, 43-80)	0.44 ± 0.14
Heavy atoms (residues 14-34, 43-80)	0.73 ± 0.16
Stereochemical quality[b]	
Ramachandran plot statistics	
Residues in most favored Ramachandran region (%)	87.4 ± 1.6
Disallowed regions [%]	0.0 ± 0.0
Unfavorable sidechain rotamers [%]	11.2 ± 1.2
Clashscore, all atoms[c]	0.0 ± 0.0
Overall MolProbity score	1.91 ± 0.05

[a] All statistics are given as mean ± S.D.

[b] Stereochemical quality according to MolProbity (http://helix.research.duhs.duke.edu).

[c] Clashscore is defined as the number of steric overlaps >0.4 Å per 1000 atoms.

Supplementary Table 2.

Site-specific substitution rates determined by maximum likelihood

**N-terminal tail**		**ICK domain 1**		**Linker**		**ICK domain 2**		**C-terminal tail**	
Site	Rate (ML)	Site	Rate (ML)	Site	Rate (ML)	Site	Rate (ML)	Site	Rate (ML)
1	1.54796	16	0.04649	47	0.41298	53	0.04649	86	26.54583
2	0.54549	17	0.51227	48	0.04649	54	0.41163	87	0.04649
3	1.0272	18	1.14064	49	0.65161	55	0.38642	88	41.4585
4	0.34311	19	0.28378	50	0.62833	56	0.67487	89	0.04649
5	0.8244	20	0.54241	51	1.94861	57	0.04649	90	0.2721
6	0.45354	21	1.02027	52	1.43003	58	0.41962	91	0.95172
7	0.25139	22	1.06503			59	0.88969	92	0.17839
8	0.04649	23	0.04649			60	0.04649	93	1.02361
9	0.04649	24	1.25779			61	4.46369		
10	1.16623	25	1.0084			62	4.33532		
11	2.15348	26	1.20354			63	3.29069		
12	0.59215	27	1.20154			64	0.88923		
13	2.67731	28	2.39168			65	4.91918		
14	1.37549	29	0.56357			66	0.76489		
15	0.43058	30	0.04649			67	0.04649		
		31	0.04649			68	0.04649		
		32	1.81822			69	23.58636		
		33	0.75351			70	0.36768		
		34	0.04649			71	0.30266		
		35	1.38722			72	41.45335		
		36	0.04649			73	0.04649		
		37	0.87793			74	2.27131		
		38	0.14624			75	1.51354		
		39	0.04649			76	10.19598		
		40	1.45829			77	5.95095		
		41	1.92611			78	4.86007		
		42	0.76378			79	9.13753		
		43	1.01329			80	5.44183		
		44	0.95717			81	0.04649		
		45	0.75918			82	0.36768		
		46	0.04649			83	6.69296		
						84	0.04649		
						85	37.55001		

Supplementary Table 3.

The ten ICK domains most similar to Xt3a domains 1 and 2 in three-dimensional sequence space[a]

Name	Accession	Distance
Domain 1		
Xibalbin 3 domain 1	JL119318.1	0.306
Xibalbin 3 domain 1	JL110103.1	0.308
Xibalbin 3 domain 1	JL112105.1	0.409
Ado1 (*Agriosphodrus dohrni*)	P58608	0.683
Mu-cyrtautoxin-As1a (*Apomastus schlingeri*)	P49268	0.699
Xt3a domain 2	JL106434.1	0.785
Mu-thomitoxin-Hme1c (*Heriaeus mellotteei*)	C0HJK5	1.010
Omega-actinopoditoxin-Mb1a (*Missulena bradleyi*)	P83588	1.217
Putative calcium channel toxin Tx758 (*Buthus occitanus*)	B8XH22	1.225
Ptu1 (*Peirates turpis*)	P58606	1.237
BmCa.1 (*Mesobuthus martensii*)	Q8I6X9	1.270
Domain 2		
Xibalbin 3 domain 1	JL119318.1	0.535
Xibalbin 3 domain 1	JL110103.1	0.540
Xibalbin 3 domain 1	JL112105.1	0.556
Xt3a domain 1	JL106434.1	0.785
Imperacalcin (*Pandinus imperator*)	P59868	0.788
Ado1 (*Agriosphodrus dohrn*)	P58608	0.865
Opicalcin.1 (*Opistophthalmus carinatus*)	P60252	0.970
Opicalcin.2 (*Opistophthalmus carinatus*)	P60253	0.979
Vejocalcin (*Vaejovis mexicanus*)	P0DPT1	0.997
Iob1 (*Isyndus obscurus*)	P58609	1.158

[a]Calcins are shown in blue

Supplementary References

1. Dulhunty AF, Curtis SM, Watson S, Cengia L, Casarotto MG. Multiple actions of imperatoxin A on ryanodine receptors: interactions with the II-III loop "A" fragment. *J Biol Chem* **279**, 11853-11862 (2004).

2. des Georges A*, et al.* Structural Basis for Gating and Activation of RyR1. *Cell* **167**, 145-157.e117 (2016).

3. Katoh K, Standley DM. MAFFT Multiple Sequence Alignment Software Version 7: Improvements in Performance and Usability. *Mol Biol Evol* **30**, 772-780 (2013).

4. Iyer KA, Hu Y, Nayak AR, Kurebayashi N, Murayama T, Samsó M. Structural mechanism of two gain-of-function cardiac and skeletal RyR mutations at an equivalent site by cryo-EM. *Sci Adv* **6**, eabb2964.
