# Supplementary material for: A bivalent remipede toxin promotes calcium release via ryanodine receptor activation
Source: Nat Commun. 2023 Feb 23;14:1036. doi: 10.1038/s41467-023-36579-w (PMC9950431; doi:10.1038/s41467-023-36579-w)
Supplement: Supplementary file 2 — Description of Additional Supplementary Files [file 41467_2023_36579_MOESM2_ESM.pdf]

Description of Additional Supplementary Files

File Name: Supplementary Data 1
Description: Multiple sequence alignment of the pancrustacean ICK peptides used to estimate the molecular phylogeny presented in Figure 5 and Supplementary Figure 12.

File Name: Supplementary Data 2
Description: Multiple sequence alignment used for the physicochemical comparison of Xt3a to functionally characterized ICK toxins shown in Figure 5.
