# Supplementary material for: A bivalent remipede toxin promotes calcium release via ryanodine receptor activation
Source: Nat Commun. 2023 Feb 23;14:1036. doi: 10.1038/s41467-023-36579-w (PMC9950431; doi:10.1038/s41467-023-36579-w)
Supplement: Supplementary file 5 — Reporting Summary [file 41467_2023_36579_MOESM5_ESM.pdf]

nature portfolio

Corresponding author(s): Mehdi Mobli

Last updated by author(s): Jan 16, 2023

Reporting Summary

Nature Portfolio wishes to improve the reproducibility of the work that we publish. This form provides structure for consistency and transparency in reporting. For further information on Nature Portfolio policies, see our Editorial Policies and the Editorial Policy Checklist.

Statistics

For all statistical analyses, confirm that the following items are present in the figure legend, table legend, main text, or Methods section.

n/a	Confirmed	
☐	☒	The exact sample size (*n*) for each experimental group/condition, given as a discrete number and unit of measurement
☐	☒	A statement on whether measurements were taken from distinct samples or whether the same sample was measured repeatedly
☐	☒	The statistical test(s) used AND whether they are one- or two-sided<br>*Only common tests should be described solely by name; describe more complex techniques in the Methods section.*
☐	☒	A description of all covariates tested
☐	☒	A description of any assumptions or corrections, such as tests of normality and adjustment for multiple comparisons
☐	☒	A full description of the statistical parameters including central tendency (e.g. means) or other basic estimates (e.g. regression coefficient) AND variation (e.g. standard deviation) or associated estimates of uncertainty (e.g. confidence intervals)
☐	☒	For null hypothesis testing, the test statistic (e.g. *F*, *t*, *r*) with confidence intervals, effect sizes, degrees of freedom and *P* value noted<br>*Give P values as exact values whenever suitable.*
☒	☐	For Bayesian analysis, information on the choice of priors and Markov chain Monte Carlo settings
☒	☐	For hierarchical and complex designs, identification of the appropriate level for tests and full reporting of outcomes
☒	☐	Estimates of effect sizes (e.g. Cohen's *d*, Pearson's *r*), indicating how they were calculated

Our web collection on statistics for biologists contains articles on many of the points above.

Software and code

Policy information about availability of computer code

Data collection	- The NMR data were acquired on a Bruker NMR spectrometer operating the Topspin v4.1.3 software (available from Bruker Biospin - https://www.bruker.com/en.html).<br>- BLAST+ v2.10.1+: used to search for genetic sequences in public databases (Camacho et al. 2009, BMC Bioinformatics 10: 421; https://blast.ncbi.nlm.nih.gov/Blast.cgi)<br>- HMMER v3.1b2: used to search for genetic sequences in public and custom databases (http://hmmer.org)<br>- The electrophysiology recordings were conducted on a Axopatch 200B and displayed using the in-house analog/digital conversion program BLM2 (available upon request).<br>- Raw fluorescence readings were converted to response over baseline using the analysis tool Screenworks 3.1.1.4 (Molecular Devices).<br>- The force responses in the muscle fibre experiments were recorded using Powerlab 4/20 series hardware (ADInstruments, Sydney, Australia).
Data analysis	- The NMR data were processed using either Topspin Topspin v4.1.3 or the Rowland NMR toolkit (available from https://rnmrtk.uchc.edu/).<br>- The NMR data were analysed using ccpnmr (available from https://ccpn.ac.uk/) version v2.4.1.<br>- Extracted NMR parameters were further used to derive structural data using the software Talos N (available from https://spin.niddk.nih.gov/bax/software/TALOS-N/) and CYANA v3.98.13 (available from https://www.las.jp/english/products/cyana.html).<br>- MolProbity (http://helix.research.duhs.duke.edu) used for evaluation of structures (Accessed 2022).<br>- MAFFT v7.304b64: used to produce sequence alignments (Katoh and Standley, 2013, Mol. Biol. Evol. 30: 772-780)<br>- MAFFT regional alignment ruby script: used to produce constrained sequence alignments (https://mafft.cbrc.jp/alignment/software/regionalrealignment.html)<br>- ModelFinder: used to determine optimal substitution model for phylogenetic analyses (Kalyaanamoorthy et al. 2017, Nature Methods

14:587-589)
- IQ-TREE v1.5.5: used to construct phylogenetic trees and perform tree topology tests (Minh et al 2020, Mol. Biol. Evol. 37: 1530-1534)
- Archaeopteryx v0.9921: used to visualise phylogenetic trees (Han and Zmasek 2009, Bioinformatics 10: 356)
- CLC Main WorkBench v7: used to examine sequences (Qiagen, Aarhus, Denmark)
- CD-HIT v4.6.5: used to filter sequences (Li and Godzik 2006, Bioinformatics 22: 1658-1659)
- GraphPad Prism v9.3.1 and Microsoft Excel (Office 365) used to calculate statistics (http://www.graphpad.com)
- SeqSpace R-package: used to cluster and visualise peptides based on their physicochemical properties (https://github.com/TS404/SeqSpace)
- For electrophysiology experiments analysis programs were Channel 2 (now depreciated; developed by P. W. Gage and M. Smith, John Curtin School of Medical Research) or Channel 3 (developed by N. W. Laver, University of Newcastle, UK). Channel 3 software can be obtained from DR Laver.

For manuscripts utilizing custom algorithms or software that are central to the research but not yet described in published literature, software must be made available to editors and reviewers. We strongly encourage code deposition in a community repository (e.g. GitHub). See the Nature Portfolio guidelines for submitting code & software for further information.

Data

Policy information about availability of data

All manuscripts must include a data availability statement. This statement should provide the following information, where applicable:
- Accession codes, unique identifiers, or web links for publicly available datasets
- A description of any restrictions on data availability
- For clinical datasets or third party data, please ensure that the statement adheres to our policy

3D coordinates are related NMR data deposited in the protein databank (PDB ID 7RZ3 [http://doi.org/10.2210/pdb7RZ3/pdb]) and Biological Magnetic Resonance Data Bank (BMRB ID 30944 [https://doi.org/10.13018/BMR30944]). All other data are available in the main text or the supplementary materials. Raw data, processing scripts and code available from the authors upon request.

Human research participants

Policy information about studies involving human research participants and Sex and Gender in Research.

Reporting on sex and gender	n/a
Population characteristics	n/a
Recruitment	n/a
Ethics oversight	n/a

Note that full information on the approval of the study protocol must also be provided in the manuscript.

Field-specific reporting

Please select the one below that is the best fit for your research. If you are not sure, read the appropriate sections before making your selection.

[x] Life sciences [ ] Behavioural & social sciences [ ] Ecological, evolutionary & environmental sciences

For a reference copy of the document with all sections, see nature.com/documents/nr-reporting-summary-flat.pdf

Life sciences study design

All studies must disclose on these points even when the disclosure is negative.

Sample size

For Cell based Fluorescence Imaging Plate Reader (FLIPR) Ca2+ assays the number of samples for each data point was 3 based on an expected effect size of > 25% and sigma of 10% giving a power of 80% at p < 0.05.

For the electrophysiology experiments, the sample size for each of the individual experimental groups in the ion channel investigation was 3 to 10 independent data sets (using internal control data from each individual RyR channel in a data set). In single RyR molecule lipid bilayer data a minimum number of 3 to 5 channels is considered sufficient to establish significance for consistent changes in channel activity, see for example: Watanabe et al: Nat Med. 2009 April ; 15(4): 380–383. doi:10.1038/nm.1942, Dulhunty et al: J Biol Chem. 2004 Mar 19;279 (12):11853-62. doi: 10.1074 Venturi et al: Biophys J. 2014 Feb 18;106(4):824-33. doi: 10.1016.

Data exclusions

Of the pancrustacean single-domain ICK sequences identified, GSCOCT00013103001.2-RA-CDS_S6D9L2 from Cotesia congregata was removed due to very long inter-cysteine loops and because its insect clade (Hymenoptera) is relatively well represented, X1X2U9 from Acyrthosiphon pisum was removed due to an unusual propeptide or very long N-terminus which could be due to frameshift, while the remipedes sequence JL112461.1 and A0A0B4UDE1 from Drosophila mojavensis were removed due to missing cysteines.

For electrophysiology experiments observations were excluded (as mentioned in detail in the manuscript Methods) where the numbers were

	were 10 to 30 times outside the range of other channels in the same experiment.<br><br>No data was excluded for any other experiments reported.
Replication	At least three replicates were generated for the electrophysiology, calcium imaging and muscle fibre experiments from which statistical significance was derived. All attempts at replication were successful other than that described under Data exclusions.
Randomization	Randomization of experimental order was carried out where possible, i.e. in the NMR data collection (order of time increments and relaxation delays).
Blinding	Blinding was not conducted. The analysis was done automatically by computer software and therefore did not require blinding.

Reporting for specific materials, systems and methods

We require information from authors about some types of materials, experimental systems and methods used in many studies. Here, indicate whether each material, system or method listed is relevant to your study. If you are not sure if a list item applies to your research, read the appropriate section before selecting a response.

Materials & experimental systems

n/a	Involved in the study	
☒	☐	Antibodies
☐	☒	Eukaryotic cell lines
☒	☐	Palaeontology and archaeology
☐	☒	Animals and other organisms
☒	☐	Clinical data
☒	☐	Dual use research of concern

Methods

n/a	Involved in the study	
☒	☐	ChIP-seq
☒	☐	Flow cytometry
☒	☐	MRI-based neuroimaging

Eukaryotic cell lines

Policy information about cell lines and Sex and Gender in Research

Cell line source(s)	SH-SY5Y (Sigma Aldrich Australia, European Cell Culture Collection) 94030304-1VL
Authentication	Cell lines were not authenticated.
Mycoplasma contamination	Cell lines were not tested for mycoplasma contamination.
Commonly misidentified lines<br>(See ICLAC register)	None

Animals and other research organisms

Policy information about studies involving animals; ARRIVE guidelines recommended for reporting animal research, and Sex and Gender in Research

Laboratory animals	Skeletal muscle tissue was obtained from back and leg muscles of euthanized mature female White NZ rabbits. Heart tissue was obtained from euthanized mature female crossbred sheep. Muscle fibres were also obtained from euthanized mice (2-6 months old, male and female).
Wild animals	No wild animals were used.
Reporting on sex	Male and female mice were used. Female sheep and rabbits were used. The effect of sex was not considered in this study.
Field-collected samples	No field collected samples were used in this study.
Ethics oversight	ANU and UQ Animal Ethics Committees.

Note that full information on the approval of the study protocol must also be provided in the manuscript.
